# Comprehensive identification of transposable element insertions using multiple sequencing technologies

**DOI:** 10.1038/s41467-021-24041-8

**Published:** 2021-06-22

**Authors:** Chong Chu, Rebeca Borges-Monroy, Vinayak V. Viswanadham, Soohyun Lee, Heng Li, Eunjung Alice Lee, Peter J. Park

**Affiliations:** 1 Department of Biomedical Informatics, Harvard Medical School, Boston, MA USA; 2 Division of Genetics and Genomics, Boston Children’s Hospital and Harvard Medical School, Boston, MA USA; 3 Broad Institute of MIT and Harvard, Cambridge, MA USA; 4 Department of Data Sciences, Dana-Farber Cancer Institute, Boston, MA USA

**Keywords:** Cancer genomics, Genome informatics, Machine learning, Software, Genomic instability

## Abstract

Transposable elements (TEs) help shape the structure and function of the human genome. When inserted into some locations, TEs may disrupt gene regulation and cause diseases. Here, we present xTea (x-Transposable element analyzer), a tool for identifying TE insertions in whole-genome sequencing data. Whereas existing methods are mostly designed for short-read data, xTea can be applied to both short-read and long-read data. Our analysis shows that xTea outperforms other short read-based methods for both germline and somatic TE insertion discovery. With long-read data, we created a catalogue of polymorphic insertions with full assembly and annotation of insertional sequences for various types of retroelements, including pseudogenes and endogenous retroviruses. Notably, we find that individual genomes have an average of nine groups of full-length L1s in centromeres, suggesting that centromeres and other highly repetitive regions such as telomeres are a significant yet unexplored source of active L1s. xTea is available at https://github.com/parklab/xTea.

## Abstract (web summary)

Identification of transposable element (TE) insertions from whole genome sequencing data remains challenging. Here the authors developed a comprehensive TE insertion detection algorithm xTea that can be applied to both short-read and long-read sequencing data.

## Introduction

Transposable elements (TEs) comprise nearly half of the human genome^1^, and their mobilization is a significant source of genomic variation and human diseases^2,3^. Although most TEs are genomic fossils that have lost their ability to mobilize, several types of TEs (L1s, Alus, and SVAs) can still mobilize via a copy-paste mechanism through RNA intermediates. Increasing evidence suggests the contribution of TEs to human development and health, such as placental development^4^ and innate immunity^5^. To date, more than a hundred TE insertions have been causally linked to Mendelian disorders and hereditary cancers, with TE impacting gene regulation through diverse mechanisms including insertional mutagenesis, premature polyadenylation, and alteration of RNA expression and splicing^3,6^.

With the availability of whole-genome sequencing (WGS) data, we have reported frequent somatic L1 insertions in some cancer types, especially in epithelial cancers, suggesting a role of TEs in tumorigenesis^7^. Subsequent studies have elaborated the role of TEs, e.g., in cancer immunity^7–12^. A recent pan-cancer analysis of ~3000 cancer genomes has identified not only numerous somatic L1 insertions, making L1 the third most frequent type of somatic SVs, but also various types of L1-mediated structural variations (SVs)^10^. In a landmark study, an SVA insertion causing exon-trapping was identified in a child with Batten disease and it led to the development of a personalized antisense-oligonucleotide drug to fix the splicing defect^13^. These studies highlight the importance of accurate TE detection for genomic medicine.

Multiple tools have been developed to detect TE insertions from Illumina paired-end short reads^7,12,14–17^. The tools include MELT^14^, which detects polymorphic inherited insertions, and TraFiC-mem^12^, which detects somatic insertions from a case/control pair. Most tools were designed to detect either germline—inherited or de novo, thus present in all cells in the body—or somatic TE insertions. One critical shortcoming of current TE analysis based on short-read data is its inability to detect TE insertions that accompany complex rearrangements or fall into highly repetitive regions, such as those within existing TE copies from the same TE subfamily or within centromeric/telomeric repeats^18–20^. Recent advances in sequencing technologies, such as PacBio and Oxford Nanopore long reads create >10–15 Kbp reads and thus allow us to reconstruct the entire sequences of inserted TEs and their flanking regions, enabling the discovery and characterization of those challenging types of TE insertions. To date, PALMER^21^ is the only tool specifically designed for TE-insertion detection from long reads.

Here, we propose a computational tool, xTea (x-Transposable element analyzer), that detects nonreference TE insertions (i.e., insertions that are not present in the reference genome) from WGS data. Rewritten from scratch for greater efficiency, it has five major improvements over the original (2012) version of Tea: (i) it has increased accuracy due to more refined filtering criteria; (ii) it identifies transduction events, both canonical and orphan; (iii) it detects a wide range of retroelement insertions, including processed pseudogene and human endogenous retrovirus (HERV) insertions; (iv) it detects both germline and somatic insertions, including mosaic insertions from very high-coverage data; and (v) it can incorporate data from multiple sequencing technologies including long-read platforms. We created a high-quality catalogue of haplotype-resolved nonreference TE insertions in an individual whose genome was extensively curated by multiple sequencing platforms. Using this annotated genome and manual inspection, we demonstrated the superior performance of xTea to existing methods for both germline and somatic insertions. Further, we performed analysis of long-read WGS data from 20 individuals. This analysis revealed complex structures and mechanisms of polymorphic insertions of various endogenous retroelements, including ‘ghost’ full-length L1s in centromeres, TE-promoted SVs, processed pseudogenes, and proviral HERV copies. xTea is available at https://github.com/parklab/xTea; its docker version is available on cloud platforms.

## Results

### Overview of xTea

**Fig. 1:**
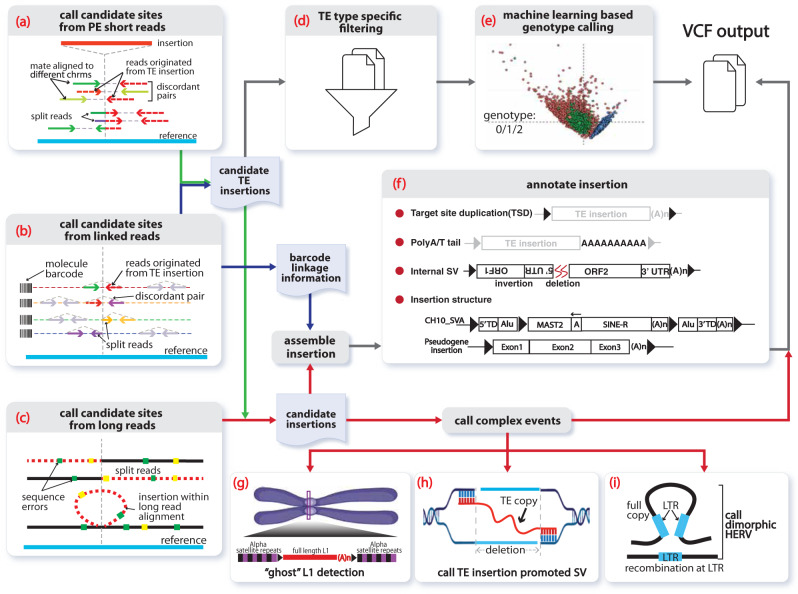
Overview of TE analysis using xTea. First, xTea identifies candidate insertion sites from three possible sets of data (or a hybrid): **a** paired-end Illumina short reads. **b** 10X Linked Reads, or **c** long reads. **d** Second, xTea filters candidate sites called from short reads for each specific transposable element (TE) type. **e** Third, for identified TE insertions, xTea uses a machine learning-based approach to call genotypes from short reads. **f** Fourth and finally, xTea annotates TE insertions and other retroelement insertions. **g** A schematic of a ‘ghost’ full-length L1 insertion detected from the centromere. **h** xTea can identify TE insertions that promote structural variation (SV) formation. **i** xTea can also identify dimorphic HERVs with given reference LTR locations.

xTea identifies nonreference TE insertions from WGS data generated using different sequencing technologies: Illumina paired-end shorts reads, 10X Linked Reads, and PacBio and Oxford Nanopore long reads (Fig. 1 and Fig. S1). It also allows hybrid TE calling when the same sample has been sequenced by more than one platform.

For standard Illumina reads, xTea utilizes two types of insertion-supporting reads—discordant paired reads and split (clipped) reads—as is standard in TE analysis (Fig. 1a). xTea, however, employs several modifications to improve accuracy and scalability. First, it considers not only the number of discordant/clipped reads but also the location of their alignment on the TE consensus sequence (Fig. S1a). Their alignment pattern must be consistent with a single breakpoint and the estimated insert size. The similar strategy is also used to filter transductions that do not manifest a consistent pattern in the flanking sequence. Second, whereas other tools derive initial candidates from discordant read pairs, xTea begins with split/clipped reads. This improves the detection of events that occur in regions close to other SVs, especially for those located within the insert size (Fig. S2). Third, xTea uses mechanistic signatures—target-site duplication and polyA tails—to further distinguish those with high confidence. Finally, xTea achieves scalability by implementing full-parallelization with multi-core support and reducing memory requirements (Fig. S3). xTea reports in the output VCF file the genotype of each insertion as predicted by a machine learning model: heterozygous, homozygous, or no insertion (Fig. 1e).

The Linked-Read technology from 10X Genomics utilizes microfluidics to partition and barcode DNA fragments from the same region, so that long-range information is embedded in the short-read data. xTea starts with the same TE-insertion detection module as for Illumina data, and then performs phased local assembly using insertion-supporting reads grouped by their molecular barcodes. It filters out candidates in regions with extremely high ‘molecular depths’ (the number of molecules whose reads encompass or fall in these regions), as this indicates highly repetitive regions. If both Illumina and Linked-Read datasets are provided for the same sample, features are extracted separately for each dataset and then merged before filtering. This early merging of raw features improves detection accuracy by increasing the signal to noise ratio more than merging of insertion candidates from each dataset. After getting raw candidates, xTea applies TE type-specific filters to create a final candidate list (see Methods).

For the PacBio and Nanopore long-read data, xTea identifies initial insertion candidates not only by examining the clipped reads with partial alignment to the flanking region of an insertion, but also those nonclipped reads that contain the entire insertion sequences (Fig. 1c). For each candidate insertion site, xTea performs local assembly of the collected supporting reads to reconstruct the full sequence of the inserted TE and flanking regions; it then annotates various features, such as subfamily, target-site duplication, polyA tail, and TE structure, prior to additional filtering (Fig. 1f). For hybrid calling with Illumina data, insertion candidates from the platforms are merged before the local assembly is performed for each site. With long-read data, xTea utilizes the fully reconstructed TE sequences to provide additional information that cannot be gleaned from short-read data. This includes identification of ‘ghost’ L1s located in centromeric regions, and identification of HERVs with nonreference proviral sequences as well as various SV-mediated TE insertions (Fig. 1g-i).

### Creation of a haplotype-resolved benchmarking dataset

A gold standard insertion set is necessary to compare xTea with existing methods and evaluate different platforms. Thus, we created a haplotype-resolved dataset of nonreference TE insertions in HG002. This HapMap sample has been extensively characterized by multiple sequencing technologies by the Genome in a Bottle consortium (GIAB)^22^ and is better than HG001 or other genomes for benchmarking. We first combined raw insertion calls, which had not been annotated for TE insertions, for HG002 from two sources: 9970 insertions (>50 bp) released by GIAB V0.6 and 15,268 insertions (>50 bp) called from a haplotype-resolved assembly^23^. Using RepeatMasker^24^, we selected insertions annotated as L1, Alu or SVA sequences, and identified high-confidence TE insertions by manually confirming that each insertion had a polyA tail and target-site duplication/deletion using the IGV^25^ browser. This is the first haplotype-resolved nonreference TE-insertion set, and will serve as a useful benchmark not only for the current study but also for other studies.

**Fig. 2:**
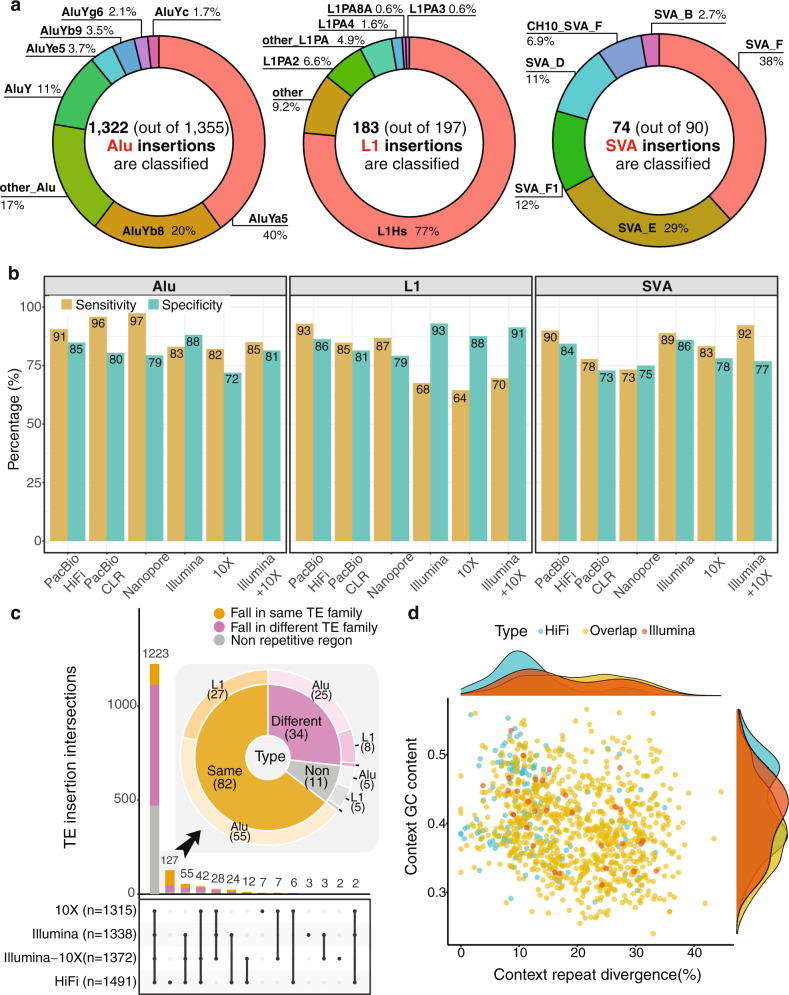
Performance comparison on sequencing data from different platforms. **a** The benchmark data (HG002) contain 1642 haplotype-resolved TE insertions, distributed as shown. Among the subfamilies, AluYa5 and AluYb8 comprise >60% of the Alus; L1Hs comprise >76% of L1s; and SVA_E and SVA_F comprise >67% of the SVAs. Notably, SVA_F1 and CH10_SVA_F make up 12% and 7%, respectively, of the SVA insertions. **b** HiFi long reads show better performance on sensitivity (91%, 93%, and 90% for Alu, L1, and SVA, respectively). Benefitting from the repeat type-specific filters, xTea shows high specificity (88%, 93%, and 86%, respectively) on short Illumina reads. Probably due to the smaller fraction of distinct molecules, 10X Linked Reads show lower specificity. **c** Detailed comparison of the number of shared TE insertions among platforms. 1223 insertions are common among all of the platforms, and 1015 (83%) of them fall in repetitive regions, out of which 261 (25.71%) fall in the same TE family while 754 (74.29%) in different TE families. 127 insertions that are only called from long reads, 116 (91.33%) fall in repetitive regions, and 82 (64.57%) are found located in repetitive regions of the same TE type. The zoomed in pie chart for the insertions exclusively called from long read shows that out of the 82 (65%) TE insertions that fall in the same context TE family, 27 (32.9%) are L1 insertions and 55 (67.1%) are Alu insertions. **d** Most of the insertions unique to long-read datasets fall in repetitive regions with low divergence rates or higher GC content, which make short-read alignment difficult.

In total, we obtained 1642 haplotype-resolved high-confidence TE insertions (1355 Alu, 197 L1 and 90 SVA insertions; Fig. S4–S5) that were present in HG002 but absent in the reference genome. Among them, we were able to identify the subfamilies for 1322 Alu, 183 L1, and 75 SVA insertions; the remaining insertions were annotated as more than one subfamily due to assembly error (Fig. 2a). For L1s, the dominant subfamily was L1Hs (>76%), although there were other polymorphic copies from the L1PA subfamily. For Alus, AluYa5 and AluYb8 comprised more than 60%. For SVAs, SVA_E and SVA_F comprised >67%, but, we also found that SVA_F1 and CH10_SVA_F (a fusion of SVA_F and the *MAST2* gene), which are difficult to detect and annotate properly because of the complex fusion structure, comprised 12 and 7% of SVA insertions, respectively.

### xTea benchmarking across multiple platforms

We applied xTea to Illumina short reads, 10X Linked Reads, PacBio High-Fidelity (HiFi) long reads, PacBio continuous long reads (CLR), and Oxford Nanopore long reads, as well as Illumina and 10X Linked Reads combined (Fig. 2b). Evaluated using the HG002 benchmark data, the most notable was the high sensitivity for L1 detection using PacBio HiFi reads: 93% for PacBio vs. 68% for Illumina, 64% for 10X, and 70% for Illumina-10X (Table S1-S3). Long reads also gave the highest sensitivity for Alu (91%, 96%, and 97% for PacBio HiFi, PacBio CLR, and Oxford Nanopore, respectively), although the improvement compared to the other platforms was smaller.

Specificity was more similar across the platforms for each TE type, with Illumina showing the highest specificity (88%, 93%, and 86% for Alus, L1s, and SVAs, respectively). Critical to the high specificity of Illumina data were additional TE type-specific filters implemented in xTea. Interestingly, 10X showed lower sensitivity and specificity than even Illumina, probably due to the smaller fraction of distinct molecules sampled in the 10X data. For long reads, false negatives were mainly due to insufficient sequencing depth, which caused the filtering of some true insertions, or the failure in later local assembly steps, e.g., assembly of SVA insertions often failed at the tandem repeat regions. With a lower per-base error rate, PacBio HiFi data performed better at the assembly step and thus had better performance overall than the PacBio CLR and Oxford Nanopore long reads.

We compared the xTea call sets from different sequencing platforms and classified each insertion according to the genomic context of the insertion sites (Fig. 2c). For long reads, we chose PacBio HiFi as the representative. The majority of TE insertions (1223 insertions) were identified by all platforms. Importantly, a large majority of these insertions (1015, 83%) were detected in repetitive regions, with 261 (26%) into the same TE family as the inserted TE and the rest into different TE families. The substantial number of cases in which a TE is inserted into the same TE family in the reference genome illustrates the difficulty of using short reads. Without the additional features implemented in xTea, many false positive insertions will be reported from those regions.

Of the 127 insertions detected only from PacBio HiFi reads, 116 (91%) insertions were found in repetitive regions, with more than half of insertions (82, 65%) found in the regions with the same TE families. Furthermore, 27 out of the 82 (33%) were L1s, which means at least 14% (27/197) of all annotated L1s were particularly difficult to detect from short reads. This explains the low sensitivity of L1 detection for other platforms (Fig. 2b). Insertions that were detected only by long reads were enriched in low divergence (from consensus sequence) repetitive genomic regions (Fig. 2d). This suggests that long-read-based detection has higher sensitivity for insertions landing in the regions with younger TE subfamilies and low mappability. The long-read-specific insertions were also enriched in GC-rich regions, consistent with inefficient PCR amplification at GC-rich regions^26^ that makes it harder for short-read-only approaches.

### Performance comparison for germline TE-insertion detection

**Fig. 3:**
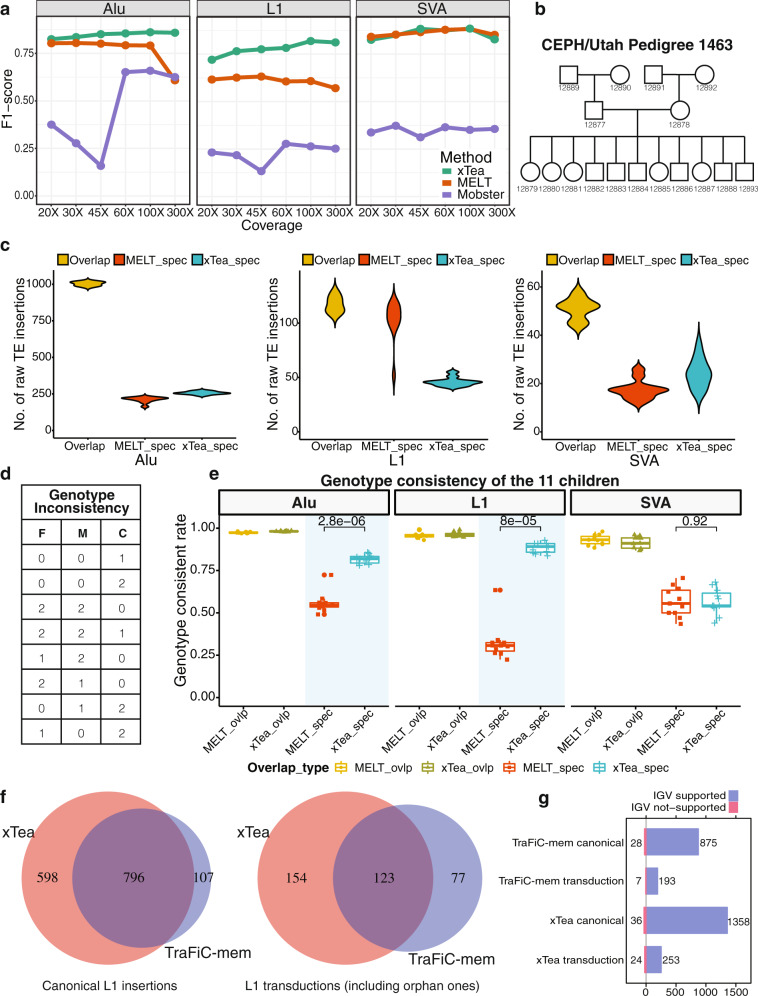
Performance comparision of xTea with MELT, Mobster, and TraFiC-mem on short reads. **a** xTea and MELT show higher F1 scores on L1, Alu, and SVA than Mobster. Compared to MELT, xTea has a higher F1 score on L1 and Alu insertions and similar performance on SVA insertions. In particular, xTea has much better performance in calling L1 insertions on all different read depths. **b** The relationship of the 17 members of the pedigree. **c** Number of overlapping, MELT-specific, and xTea-specific Alu, L1, and SVA insertions in the 11 children. **d** The defined genotype inconsistency, where ‘F’, ‘M’, and ‘C’ indicate the genotype of father, mother, and child, respectively, and ‘0’, ‘1’, and ‘2’ represents reference homozygous, heterozygous, and homozygous alternate, respectively. **e** Insertions overlapping between xTea and MELT show similar genotype consistency. For nonoverlapping ones, xTea performs much better on Alu and L1 than MELT. For the boxplots, the box demarcations represent the 25, 50, and 75th percentiles, and the whiskers extend from the box to the largest and smallest data points at most 1.5 times the interquartile range away from the median. **f** Overlaps between xTea and TraFic-mem on somatic canonical L1 insertions and L1 transductions for 15 colon samples. **g** Manual inspection of all the L1 insertions detected by both xTea and TraFiC-mem through IGV screenshot. For TraFiC-mem, 28 canonical L1 insertion, and 7 transductions are checked as false positive; for xTea, 36 canonical L1, and 24 transductions are ‘validated’ as false positives.

We first compared the performance of xTea with MELT^14^ (v2.1.5) and Mobster^15^ for germline TE-insertion detection and with TraFiC-mem^12^ for somatic TE-insertion detection. MELT has been widely adopted by several projects, such as the 1000 Genomes Project and the gnomAD-SV database^27^. For our evaluation, we realigned the original ~300X Illumina paired-end WGS data for HG002^28^ to hg38 with BWA-MEM^29^, and downsampled to various sequencing depths (20X-100X). Both MELT and xTea showed much better performance than Mobster in detecting all the three types of TE insertions (Fig. 3a; S6 for detailed sensitivity and specificity), while xTea showed better performance in detecting L1 and Alu insertions than MELT across all sequencing depths tested. The performance difference increased as the depth of sequencing increased, with MELT reporting more false positives. For SVA, MELT, and xTea showed comparable performance. (For MELT, there are three possible sets of variants: raw output, PASS calls, and genotype calls. We used those genotyped as ‘0/1’ and ‘1/1’ since those calls gave the best results; PASS calls had low sensitivity, whereas the raw output had low specificity).

We next compared genotyping accuracy of xTea and MELT using Illumina WGS data from a large family (CEPH pedigree 1463) with 17 members across three generations^30^ (Fig. 3b). We first ran MELT and xTea on each sample to identify Alu, L1, and SVA insertions. Comparison of the call sets on the 11 children shows that a large fraction of the Alu and SVA candidates are shared between MELT and xTea (Fig. 3c). For Alu, 960 calls are shared while 200 are MELT-specific and 280 are xTea-specific on average; for SVA, 50 calls are shared while 18 are MELT-specific and 27 are xTea-specific. For L1s, the number of MELT-specific calls are much greater and is highly variable.

To determine the quality of the MELT-specific and xTea-specific calls, we measured genotype consistency by checking parental and grandparental genotypes of each insertion found in a child (Fig. 3d,e; S7b,c for individual level). Given a low rate of de novo insertions (1 out of ~20 births)^31^, we considered insertions that violate the Mendelian inheritance as insertions with inconsistent genotypes. For example, for a given TE insertion, if both parents are homozygous (genotype ‘1/1’ or 2), then no insertion (genotype ‘0/0’ or 0) or a heterozygous insertion (genotype ‘0/1’or 1) in the child was considered as inconsistent; other inconsistent genotypes are listed in Fig. 3d. Not surprisingly, those TE insertions overlapping in the two candidate sets showed high levels of genotype consistency (Alu: 0.97 vs 0.98; L1: 0.96 vs 0.96; SVA: 0.93 vs 0.91, all for MELT and xTea, respectively). For the caller-specific candidates, xTea-specific insertions showed a dramatically higher genotype consistency for L1 and Alu than MELT-specific insertions (0.82 vs 0.56 for Alu; 0.88 vs 0.32 for L1). For SVA, genotype consistency was comparable between the two methods. Checking for genotyping consistency not only between child-parent but also parent-grandparent pairs showed even better performance for xTea in detecting Alus and L1s (Fig. S7d).

To further evaluate the performance of xTea in genotype calling, we evaluated the performance of xTea using a PCR benchmark dataset^32^. This dataset reported PCR validation results at 145 Alu sites for 90 samples from the 1000 Genomes project including 45 high-coverage (~30X) samples. When we compare the genotypes called by xTea with the PCR data for these 45 samples, we find that the results are highly consistent, except for the 11 sites that xTea genotyped as heterozygous (0/1) but the PCR data showed homozygous (1/1) (Table S4). These 11 genotypes may be incorrect predictions by xTea, but it is also possible that the genotypes from PCR are imprecise.

For long reads, we first compared xTea with PALMER on the haplotype-resolved HG002 benchmark data. We used two groups of PALMER calls in the comparison: “raw”, which are all the reported calls, and “HC”, which are high-confident calls that utilize additional filtering criteria. The results (Fig. S8) show that xTea outperforms PALMER on both sensitivity and specificity across all three TE families. Notably, PALMER reports many false positives resulting in low specificity for Alu (0.4 vs 0.85 for PALMER and xTea, respectively) and L1 (0.33 vs 0.86). The PALMER paper describes a manually inspected call set of 203 L1 insertions for the PacBio CLR data from a different sample, HG001. With xTea run on the PacBio HiFi data for this sample, we obtain 208 L1 insertions. Between the two call sets, there are 168 common insertions, as well as 40 xTea-specific and 35 PALMER-specific insertions (Fig. S9).

In theory, one could attempt to identify the same TE-associated breakpoints by a general-purpose SV caller. Therefore, we also compared xTea with general-purpose SV callers on both short- and long-read data. For short reads, we evaluated DELLY^33^ and Manta^34^ on the same HG002 benchmark data (Fig. S10). The results indicated two major limitations for general SV callers in TE-insertion calling: much lower sensitivity (Alu: 0.12, 0.37 vs 0.83; L1: 0.2, 0.53 vs 0.68; SVA: 0.46, 0.58 vs 0.89, all for DELLY, Manta, and xTea, respectively) and absent or incorrect annotation of TE insertions. The low sensitivity is due to the fact that a general SV caller searches for two clusters of discordant read pairs when a TE insertion has a cluster only on one side. In terms of annotation, TE insertions are often marked as translocations (to a lesser extent, duplications, inversions, or other types) because TE-specific features are not considered. For long reads, we ran Sniffles^35^, CuteSV^36^ and SVIM^37^ on the PacBio HiFi reads from the same HG002. These callers had much lower sensitivity (Alu: 0.87, 0.85 vs 0.91; L1: 0.76, 0.53 vs 0.93; SVA: 0.58, 0.33 vs 0.9, all for CuteSV, Sniffles, and xTea, respectively) or much higher false positive rate (SVIM reported >10 times more SVs) compared to xTea (Fig. S11-S12). Some long-read SV callers, including Sniffles, attempt to assemble insertions. Thus, it is also possible to run such an SV caller and then use RepeatMasker to annotate TE insertions. However, this is only limited to simple and canonical Alu and L1 insertions; other repeats such as SVAs and transduction or complex events cannot be identified (Table S5). Overall, all these results underscore the importance of transposon-specialized callers, such as xTea, that assemble and annotate TE insertions to achieve high sensitivity and specificity.

### Performance comparison for somatic L1 insertion and transduction detection

We compared the performance of xTea with TraFiC-mem^12^ (Transposon Finder in Cancer), an algorithm used in a recent analysis of L1-mediated rearrangements in cancer by the International Cancer Genomics Consortium.^10^ We examined their accuracy in detecting somatic L1 insertions, including those with transduction, from 15 colon samples (those likely to have the highest rate of L1 insertions) and their matched blood samples. xTea identified a total of 1671 somatic L1 insertions (1394 canonical, 277 with transduction), whereas TraFiC-mem detected a total of 1103 L1 insertions (903 canonical, 200 with transduction). The percentage of the shared calls is 55% (919/1671) for xTea and 83% (919/1103) for TraFiC-mem. Through manual inspection of each insertion candidate using the IGV browser, we confirmed that 96 and 97% of xTea and TraFiC-mem calls showed insertion-supporting signal (both sides discordant pairs, split reads, target-site-duplication, and polyA tail) (Fig. 3f-g). With the similar precision level, xTea predicted significantly more L1 insertions, indicating a higher sensitivity for xTea compared to TraFiC-mem.

### Structure of L1 insertions and discovery of centromeric L1s by long-read analysis

**Fig. 4:**
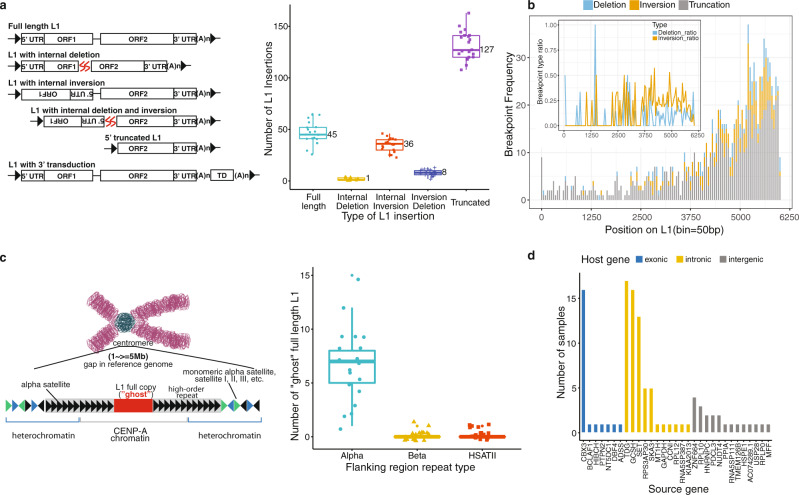
L1 and processed pseudogene insertion from long reads. **a** Left is the scheme of different types of L1 insertions, including the full-length L1, L1 with internal deletion, L1 with internal inversion, L1 with both internal deletion and inversion, 5’ truncated L1 and L1 with transduction. The boxplot at right shows the number of detected L1 insertions of those 5 types for the 20 long-read samples. **b** We checked the internal deletion, internal inversion, and truncation breakpoint frequency by position on L1 for all the detected non redundant L1 insertions. More internal deletions, internal inversions, and 5’ truncations happen at the tail side. The inset figure shows the normalized ratio for internal deletion and inversion by position, which indicates higher inversion rates than deletion from position ~4000 to the end. **c** Left is the scheme of centromere full-length L1. Right is the number of centromere ‘ghost’ full-length L1 copies per sample. On average per sample, we detected about 7, 1, and 1 full-length L1 copies flanked with Alpha, Beta, and HSATII satellite repeats, respectively. **d** 31 pseudogene insertions were detected from the 20 samples, including 7, 11, and 13 pseudogene insertions falling within exonic, intronic, and intergenic regions, respectively. 20 (64.52%) of the insertions are only detected in one sample. For the boxplots in **a** and **c**, the box demarcations represent the 25, 50, and 75th percentiles, and the whiskers extend from the box to the largest and smallest data points at most 1.5 times the interquartile range away from the median.

Multiple mechanisms, such as target-primed reverse transcription and twin priming, drive the creation of L1s with heterogeneous structures in the human genome^38^. However, the structural landscape of polymorphic L1 insertions has remained largely unknown due to the limitation of short reads. Long-read sequencing provides a powerful means to fill this knowledge gap. We analyzed long-read WGS data of 20 individuals (Table S6) from 5 different human populations, released in two recent studies^39–41^. Using the long-read mode of xTea, we identified 1,160 polymorphic L1 (285 full length and 875 5’-truncated L1) insertions and constructed their entire sequences (Fig. S13). Each individual had an average of 217 polymorphic L1 insertions with the following structures: 45 (21%) full-length L1s, 36 (17%) L1s with internal inversion, 1 (0.46%) L1 with internal deletion, 8 (3.7%) L1s with both internal deletion and inversion, and 127 (59%) 5’ truncated L1s (Fig. 4a). In general, the tail side contained more internal deletions, internal inversions, and 5’ truncations (Fig. 4b), with a higher inversion rate than deletion rate toward the tail (>4000 bp) (Fig. 4b, inset).

Despite the high assembly quality of the human reference genome, there remains several hundreds of gaps, especially in the centromeres^42^. Enriched with highly repetitive satellite repeats associated with epigenetic regulation^43^, centromeres may be an important source of retrotransposition-competent full-length L1s. We developed and applied a new approach to the 20 long-read-sequenced genomes to identify such L1s in centromeric regions (Fig. 4c). Briefly, we collected long reads that spanned a reference full-length L1 with both sides clipped at the two flanking sides of the L1; we then grouped the collected long reads based on flanking sequences, assembled each cluster of flanking sequences into a contig sequence, and annotated the assembled contig using RepeatMasker to determine whether the insertion falls within centromeric repeats (a detailed schematic is shown in Fig. S14). This analysis yielded an average of nine groups full-length L1 insertions within the centromere regions per genome: seven within Alpha satellite repeats, one within Beta satellite repeats, and one within HSATII repeats (Fig. 4c). One of the centromeric L1s we identified was reported in a recent annotation of the high-quality assembly of human chromosome X^19,20^ (Table S7). In addition, we ran xTea on the CHM13 HiFi long reads and constructed 13 groups of potential centromere full-length L1s. To validate them, we annotated all the potential centromere full-length L1s from CHM13 telomere-to-telomere assembly v1.0 (https://github.com/nanopore-wgs-consortium/CHM13), and found 114 full-length L1s that can be clustered into 18 groups (Table S8). The 13 groups constructed from xTea together with their flanking regions could be well aligned to the assembly.

### HERV insertions, pseudogene insertions, and TE-mediated rearrangements

Endogenous retroviruses (ERVs) are derived from exogenous retroviruses that are integrated into the host genomes. A full-length (proviral) ERV is comprised of an internal protein-coding region flanked by two long terminal repeats (LTRs). Several human ERV (HERV) families have been associated with several diseases, including several cancers, neurological, and autoimmune diseases^44–48^. Because of the sequence homology, LTR-LTR recombination will result in the deletion of the internal coding sequence. For the same proviral HERV, if recombination only happens in some samples, it will result in “dimorphic HERV”^49^, where the reference genome is a solo LTR but may be proviral HERV in individuals. Many of these complex events of different HERV subfamilies, for instance HERV-K and HERV-H, have been reported from short paired-end reads analysis^49^. However, short reads can be used to check the two tail sides of an event, but they do not provide the full structure; short reads also do not provide information for those events in repetitive or complex regions.

**Fig. 5:**
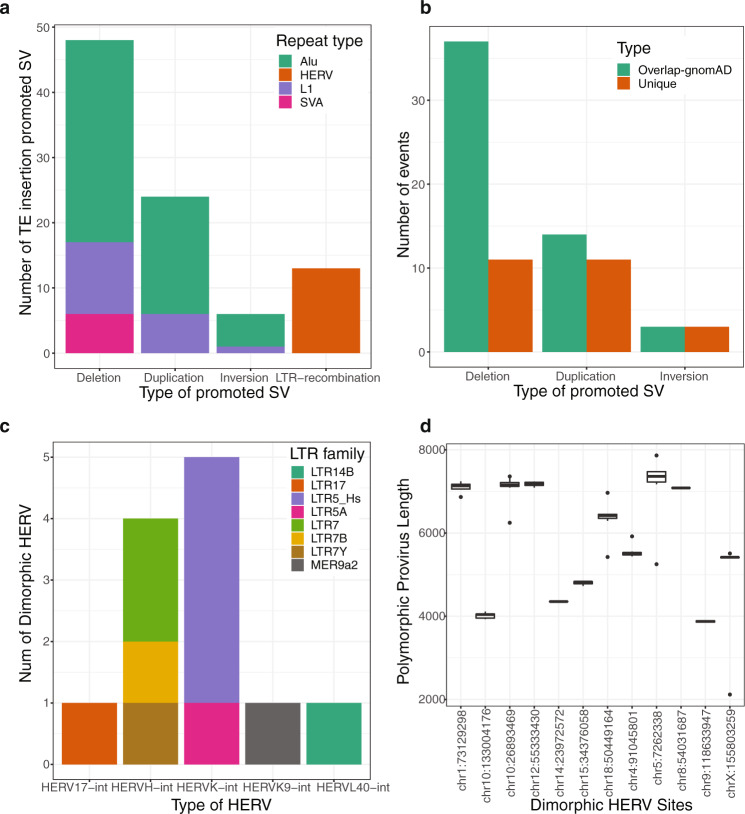
Structural variants mediated by TE insertions. **a** xTea called out several structural variants promoted by insertions of different types of TEs from the 20 long-read samples: 48 (31 Alu, 11 L1, and 6 SVA) deletions, 24 (18 Alu and 6 L1) duplications, and 6 (5 Alu and 1 L1) inversions. In addition, xTea called out 12 LTR recombination-caused dimorphic HERV events. **b** Compared to gnomAD-SV, more than 15, 40, and 50% of the TE-mediated deletions, duplications and inversions are novel. Each of these complex SVs are only called as one type of simple events, although the breakpoint positions are the or close to each other. **c** 6 HERV-K, 4 HERV-H, 1 HERV-L, and 1 HERV-W dimorphic HERV events are called out. Compared to a previously reported call set, 50% of the HERV events are novel. **d** Internal deletions occur in some dimorphic HERV insertions. Boxplot of each site shows the length of the HERV copy among different samples. While some copies are of full length (~7200 bp), some copies are only ~4000 bp although the front and tail parts exist. For the boxplots, the box demarcations represent the 25, 50, and 75th percentiles, and the whiskers extend from the box to the largest and smallest data points at most 1.5 times the interquartile range away from the median.

Here, we ran xTea on the 20 long-read samples and detected 12 HERV insertion loci with internal proviral sequences by screening genomic regions annotated to have solo long terminal repeats (LTRs) without the internal proviral sequence in the reference genome (see Methods). Specifically, xTea detected six HERV-K, four HERV-H, one HERV-L, and one HERV-W proviral sites (Fig. 5c). Our analysis of only 20 genomes detected all six HERV proviral insertion loci that were previously reported from an Illumina WGS analysis of 279 individuals from very diverged populations, as well as six more novel loci^49^. This suggests that more polymorphic proviral HERVs may be discovered through analysis of more genomes. We also found internal deletions within the HERV insertions (Fig. 5d). For example, a HERV located at chr10:133004176 (hg38) had a ~3 kb internal deletion (full length is ~7 kb) across all 20 individuals. With short-read-only data, such deletions are unlikely to be fully annotated^49^.

Processed mRNA of genes can mobilize to create polymorphic pseudogene insertions, potentially causing rare diseases^50,51^. Somatic pseudogene insertions also occur in many human cancers^10,52^. With the long-read data, we detected and constructed the entire sequences of 31 pseudogene insertions. Among these, 7, 11, and 13 insertions were found in exonic, intronic and intergenic regions, respectively (Fig. 4d). Twenty (65%) of the insertions were detected in a single sample, suggesting that a large portion of pseudogene insertions are evolutionarily recent events. Of all the detected insertions, 18 (58%) insertions were not reported in a previous study^53^. Our results are consistent with a recent study^54^ based on long-read de novo assembly that reports a higher rate of processed pseudogene insertions in humans.

We also identified 78 SVs that were formed as a result of double strand breaks induced during TE transposition: 48 deletions (31 Alu, 11 L1, and 6 SVA), 24 duplications (18 Alu and 6 L1), and 6 inversions (5 Alu and 1 L1) (Fig. 5a). Among them, 15% of deletions, 40% of duplications, and 50% of inversions were not reported in the latest SV database, gnomAD-SV (v2.1.1)^27^ (Fig. 5b); the rest were called only as simple events. As our results are from 20 individuals (compared to ~15,000 in gnomAD-SV), far more TE-mediated complex SVs are likely to be present in the population.

## Discussion

We presented a computational method, xTea, to identify, genotype, and annotate both germline and somatic TE insertions in WGS data. Whereas many analyses have focused on the reference genome TE copies^55,56^, it is important to annotate polymorphic TE insertions, which are generally more recent and may play an important role in regulating the host gene expression. As the number of short-read WGS datasets continues to grow rapidly, xTea can be used to build a more comprehensive database of polymorphic TE insertions and trace their source elements through transduction events, as well as investigate the role of somatic insertions in cancer genomes.

With a high-quality benchmark dataset and a large pedigree dataset, we demonstrated that xTea outperforms MELT in identifying and genotyping germline insertions. xTea also has much higher sensitivity with comparable precision in identifying somatic L1 insertions than TraFiC-mem. However, xTea may also miss some cases when there is not enough clipped reads support, especially for tumor samples with low purity; in such cases, users need to adjust the parameters accordingly.

Another key feature of xTea is its capability to analyze long-read data. Not surprisingly, xTea obtained higher sensitivity with long reads than with short reads at comparable specificity, especially for insertions in low-diverged repeats and GC-rich regions. Our examination of 20 genomes led to the creation of the most comprehensive catalogue of polymorphic retroelement insertions. The long reads also allowed us to resolve the insertional structures of not only L1s but also pseudogenes, TE-insertion-mediated SVs, and HERV loci containing proviral sequences. Although still expensive for routine sequencing of samples, PacBio and Nanopore sequencing of a larger cohort will be necessary for a better understanding of the repetitive elements. A recent Nanopore dataset of 3622 Icelanders^57^ is one such example. Compared to the original PacBio CLR and Nanopore reads, PacBio HiFi reads with their low error rate showed better performance in detecting the breakpoints precisely, assembling the TE copies, and identifying internal mutations within the TEs, especially for low-coverage data for which error correction is not easy to perform. One advantage for Nanopore over PacBio is that the DNA methylation state can be directly detected from the reads, thus providing the opportunity to check the epigenetic silencing of TE.

One of the interesting results in our study is the characterization of TEs that landed in centromeres, but more work is needed to understand their function, if any. When identifying such ‘ghost’ L1s, we used stringent filters to call the candidates, but further refinement may be needed. For example, on the one hand, false positives may be reported if the flanking sequences are homologous to centromeric satellite repeats; on the other hand, false negatives may result if none of the two flanking regions of a full-length centromere L1 contain satellite repeats. We have focused on full-length L1 copies to keep the specificity high, but truncated L1s and other types of TEs can also exist in centromeres. Recent efforts for end-to-end chromosome assemblies^19,20^ constructed full centromeric sequences and will help uncover the biological significance of centromeric TEs.

## Methods

### Procedures for TE-insertion identification

#### Illumina paired-end short reads

For each input bam/cram file, xTea first extracts the information on sequencing depth, insert size, and read length. Unless specified by the user, xTea automatically determines insertion calling thresholds, such as the number of insertion-supporting clipped reads and the number of discordant reads, based on the sequencing depth. If an insertion falls in a repetitive region that comprises the same TE family as the candidate insertion and the reference repeat has a lower divergence rate from the RepeatMasker consensus than a specified threshold, this candidate is removed. xTea improves specificity by examining the patterns of insertion-supporting clipped sequences and discordant reads mapped to the TE consensus sequences (Fig. S1a): the supporting reads should not be scattered across the consensus but instead form one cluster (c1) for 5’-clip reads and another cluster (c2) for 3’-clip reads; the mates of 3’ and 5’ discordant reads should form two distinct clusters (d1 and d2). The distance between c1 and d2 and between c2 and d1 must be less than the average insert size ±3´standard deviation. Additionally, xTea detects TE insertions with transduction and target-site deletions. These events often show clusters of clipped and discordant mate reads on only one side when the reads are mapped to TE consensus sequences (for transduction, reads supporting the other side of the breakpoint do not originate from TEs; for target-site deletion, reads from different breakpoints originate from genomic regions far from each other). xTea initially identifies all candidates with consistent c1 and d1 clusters or consistent c2 and d2 clusters. For candidates with support on only one side of the breakpoint, xTea collects discordant reads from the other side of the breakpoint to trace the source TE for transductions (see Fig. S1a) or examines read depth patterns to find the other breakpoint for target-site deletions.

#### 10X linked reads

xTea detects initial candidates using the same procedure as above, and, after grouping reads according to the barcode for each haplotype, performs local assembly for each read group (Fig. 1b, S1b). xTea first filters out genomic regions with extremely high molecule coverage to improve processing speed (default: >250X). Then, for each candidate site, xTea collects reads mapped near the insertion and creates three subgroups: reads that belong to haplotype 1, reads that belong to haplotype 2, and unphased reads. For each subgroup, xTea performs local assembly and aligns the flanking reference sequence to the assembled contig. If the two flanking regions are well aligned but far apart from each other, the sequence between the two flanking regions is identified as the insertion sequence. Local assembly and mapping procedure are performed for all reads first and then the three subgroups in the order listed. When an insertion is predicted in a subgroup, xTea will not examine the remaining subgroups. xTea maps the insertion sequence to TE consensus sequences to annotate the TE family. Overall, xTea reports not only the exact TE-insertion breakpoints but also the assembled insertion sequences and haplotypes to which each insertion belongs. For some candidates, xTea fails to reconstruct the insertion sequence due to low sequencing depths or genomic complexity of insertion sites.

#### PacBio and Oxford nanopore long reads

For short reads, clipping mostly occurs at the same position across different reads. In contrast, long reads, due to a higher sequencing error rate, are clipped at more variable positions around the breakpoint (Fig. 1c, S1c). xTea therefore groups clipped reads within a specified distance (by default: 75 bp) and removes the group if the standard deviation of the distance between the clipped coordinate of each read and the median coordinate of clipped reads is greater than a threshold (default: 45 bp). In subsequent steps, the median coordinate is used as the site of the insertion candidate. This strategy has also been used by Sniffles^35^ in calling SVs from long reads. For each candidate site, xTea collects all reads clipped close to the site, as well as reads with internal insertion breakpoints close to the site (calculated from the ‘CIGAR’ field of the read alignments), followed by local assembly of the collected reads using wtdbg2^58^. The 5’ and 3’ flanking reference sequences are then aligned to the assembled contigs to identify insertion sequences. Finally, each insertion sequence is aligned to the TE consensus sequences to annotate the inserted TE family. For L1 and SVA insertions, xTea calls transduction events by realigning the clipped sequences to the flanking sequences of all reference and polymorphic full-length copies. If the clipped sequence is uniquely aligned to the flanking sequences, xTea annotates the full-length TE as the source TE of the insertion. xTea also applies a breakpoint refinement step by using the assembly supported breakpoints to replace the cluster central site breakpoints. Steps are run in parallel whenever possible to improve computational efficiency.

#### Hybrid data from more than one platform

In general, analysis of hybrid data from different sequencing platforms combines the advantages of each platform to improve detection performance (Fig. 1a). For datasets consisting of Illumina short reads and 10X Linked Reads, xTea merges the clipped reads and discordant reads from the two platforms to identify initial insertion candidates. In addition, local assemblies using Linked Reads are performed to construct the insertion for each candidate. For datasets consisting of Illumina short reads and long reads, candidates from Illumina short reads are merged with candidates from long reads, and local assembly is conducted using long reads for each site.

### Machine learning-based TE-insertion genotyping for short reads

As described in the previous section, several features, e.g., the number of clipped reads, are used to detect a TE insertion. Similarly, these features can also be used to determine the genotype of an insertion. The key observation is that for no insertion/reference homozygous (0/0), heterozygous (0/1), and homozygous (1/1), the quantity of the features is different. Here, we considered TE-insertion genotyping as a classification problem for which we can train a machine learning model^59^. As general machine learning approaches, the genotyping module consists of three parts: feature extraction, model training and genotype prediction. We extracted 14 features for each candidate (Fig. S15b), normalizing them by the average sequencing depth where needed. We prepared a training set by taking high-confidence calls (supported by both sides of clipped and discordant reads, a polyA tail, and a target-site duplication) from unaffected individuals in WGS data from ~1800 trio families. Specifically, if a call was made in only one parent and no supporting clipped reads were present in the other parent, it was labeled as heterozygous (0/1). If a call did not have supporting clipped reads in any parent, it was considered a false positive and labeled *no* insertion/reference homozygous (0/0). If a call was made in both parents, the ratio between discordant and all (discordant + concordant) reads was >0.85, and no fully mapped reads at the breakpoints, it was labeled as homozygous (1/1). We identified a total of 334,609 homozygous, 1,070,309 heterozygous, and 18,959 no insertion sites. Using 70% of the genotyped sites as training data, we trained a random forest model. Applying this model to the testing data (30%), we obtained 99.7% accuracy (Fig. S15a;15c). The importance scores of the features are shown in Fig. S15b.

### Somatic TE-insertion detection

We first run xTea on the case sample to identify all candidate TE insertions, both somatic and germline. Unlike in germline variant calling, xTea considers the clonality of insertions (e.g., tumor purity) in determining detection parameters. Next, for each candidate TE insertion in the case sample, we check the number of supporting clipped reads and discordant pairs in the control sample. We report an insertion as somatic if there is no or few (criteria adjusted automatically based on read depth) supporting reads. Without matched control, it is more difficult to detect somatic TE insertions, especially if they are present in a small fraction of cells; nonetheless, xTea can generate a list of candidates using a lower threshold for the number of supporting reads and use visual inspection and experimental validation to remove false positives.

### Annotation of deletions and inversions within L1s from long reads

For each L1 insertion detected from long reads, we align the assembled contig sequence to the L1 consensus sequence with minimap2^60^. If the contig is fully aligned without clipping, we classify it as a full length or 5’ truncated copy based on the insertion length. If the contig is aligned with clipping, then we realign the clipped sequence. If the distance between the two aligned parts is sufficiently large (default: >20 bp), we annotate the insertion as having an internal deletion. If the two parts are aligned in different orientations, we label the insertion as having an internal inversion.

### Identification of ‘ghost’ full-length L1s from long reads

To identify full-length L1s within centromeric repeats that conventional approaches cannot detect, we first extracted all full-length reference L1 copies based on the RepeatMasker annotation. Then, we collected all reads that align to these full-length L1s with clippings at both flanking regions (Fig. S14). We observed that if reads come from the same ‘ghost’ copy, then the left/right clipped parts will be aligned close to each other, as they are sequenced from the same flanking region. But they will not align to clipped reads from a different ‘ghost’ copy. In other words, flanking region similarity could be used to cluster the collected reads. Based on this observation, we aligned the clipped sequences to each other and clustered them based on sequence similarity. For each cluster, we performed local assembly to get the L1 and its two flanking sequences. Next, we ran RepeatMasker on the two flanking sequences and annotated it as a ‘ghost’ L1 if any flanking sequence is masked as Alpha, Beta, or HSATII centromeric satellite repeats. To improve specificity, we examined full-length L1s from centromeres only, although other highly repetitive regions, such as telomeres, may also host them.

### Detection of pseudogene insertions from long reads

First, we create a fasta file that includes all exon sequences based on the GENCODE gene annotation (v33)^61^. Then, we align all the exon sequences to the local assembled insertion sequences with BWA^29^. If an assembled insertion sequence is covered by concatenated exons of one gene and there is a polyA/T tail detected at the end of the insertion sequence, then this insertion is considered a processed pseudogene insertion of that specific gene. Note that some exons are short and may be multiply mapped. To filter out false positives, we require the exon sequence to be uniquely mapped (by default, with minimum mapping quality 30).

### Detection of TE-insertion-mediated SVs from long reads

xTea detects different types of SVs (deletions, inversions, and duplications) mediated by nonreference TE insertions. Detection of such events is challenging and requires additional considerations. For example, a TE-insertion-mediated deletion will have left-clipping and right-clipping positions much farther apart than the size of target-site duplication (TSD) in canonical TE insertions. To detect this event, we first collect all the left breakpoints at which reads are left-clipped (we describe the procedure for the left side for simplicity; equivalent steps are carried out for the right side). For each breakpoint, we extract its right-flanking region from the reference genome. Second, for each breakpoint, we carry out local assembly for all its left-clipped parts. Third, we align all the flanking regions of the ‘left’ breakpoints to the ‘right’ assembled contigs. For two breakpoints A (left breakpoint) and B (right breakpoint), if the flanking region of A is aligned to the assembled contig of B and vice versa, then we designate A and B as paired breakpoints.

For each pair of breakpoints, we infer the type of SV represented by the breakpoints based on how the insertion sequence is aligned to the TE consensus sequence. The SV is a TE-insertion-mediated deletion if the internal sequence is fully mapped and the two flanking regions are aligned apart from each other on the reference genome. The SV is a TE-insertion-mediated inversion event if the internal sequence is partially aligned on the consensus, the clipped part is well aligned to the reference genome with different orientation, and the two flanking regions are aligned exactly to the two sides of this inverted region on the reference genome. The SV is a TE-insertion-mediated duplication event if the middle part of the internal sequence is aligned to TE consensus, the two side clipped regions are aligned to the same region on the reference genome, and the two flanking regions are aligned exactly to the two sides of this region on the reference genome.

### Detection of LTR recombination-associated dimorphic HERV copies

As shown in Fig. S16, we first extract all the annotated reference LTR repeats from RepeatMasker. Second, for each extracted LTR repeat, we check whether reads are clipped aligned at the boundary. If so, we collect all these clipped reads for each LTR repeat and perform local assembly. Third, we align the two flanking regions of the LTR repeat to the assembled contigs. If both of the flanking regions are well aligned, we extract the middle part as the candidate HERV copy. Finally, we align the LTR repeat to the candidate copy, and if it is aligned to both ends of the candidate copy, and also the middle part is masked as HERV by RepeatMasker, we designate the site as a dimorphic HERV copy driven by LTR recombination.

### Reporting summary

Further information on research design is available in the Nature Research Reporting Summary linked to this article.

## Supplementary information

Supplementary Information

Peer Review

Reporting Summary

## Data Availability

Sequencing data of sample HG002 were downloaded from The Genome in a Bottle Consortium (https://docs.opendata.aws/giab/readme.html). The Platinum Genomes pedigree data were downloaded from dbGaP (https://www.ncbi.nlm.nih.gov/gap/) study phs001224.v1.p1. Information on accessing raw data of the 15 colon cancer samples can be found at https://docs.icgc.org/pcawg/data/. The long-read sequencing data were downloaded from the International Genome Sample Resource (IGSR) at https://www.internationalgenome.org/data/; AWS Open Dataset from https://github.com/human-pangenomics/hpgp-data; and studies NCBI (https://www.ncbi.nlm.nih.gov/bioproject): PRJNA300843, PRJNA300840, PRJNA288807, PRJNA339722, PRJNA385272, PRJNA339719, PRJNA339726, PRJNA323611, PRJNA481794, PRJNA480858, and PRJNA480712. The CHM13 data were downloaded from Telomere-to-telomere consortium (https://github.com/nanopore-wgs-consortium/CHM13). Gene annotation data were downloaded from GENCODE (https://www.gencodegenes.org/human/). RepeatMasker annotation data were downloaded from https://www.repeatmasker.org/species/hg.html. The data supporting the findings of this study are available from the corresponding authors upon reasonable request.

## Code availability

Source code for xTea is available for download at https://github.com/parklab/xTea (10.5281/zenodo.4743788).
