# Supplementary material for: Comprehensive identification of transposable element insertions using multiple sequencing technologies
Source: Nat Commun. 2021 Jun 22;12:3836. doi: 10.1038/s41467-021-24041-8 (PMC8219666; doi:10.1038/s41467-021-24041-8)
Supplement: Supplementary file 1 — Supplementary Information [file 41467_2021_24041_MOESM1_ESM.pdf]

Supplementary file for:

Comprehensive identification of transposable element insertions using multiple sequencing technologies

Chong Chu[1], Rebeca Borges Monroy[2,3], Vinay Viswanadham[1], Soohyun Lee[1], Heng Li[1,4], Eunjung Alice Lee[2,3]* and Peter J. Park[1]*

[1]Department of Biomedical Informatics, Harvard Medical School, Boston, MA 02115, USA.

[2]Division of Genetics and Genomics, Boston Children's Hospital and Harvard Medical School, Boston, MA 02115, USA.

[3]Broad Institute of MIT and Harvard, Cambridge, MA 02142, USA.

[4]Department of Data Sciences, Dana-Farber Cancer Institute, Boston, MA 02142, USA.

*to whom correspondence should be addressed. E-mail: peter_park@hms.harvard.edu or ealice.lee@childrens.harvard.edu

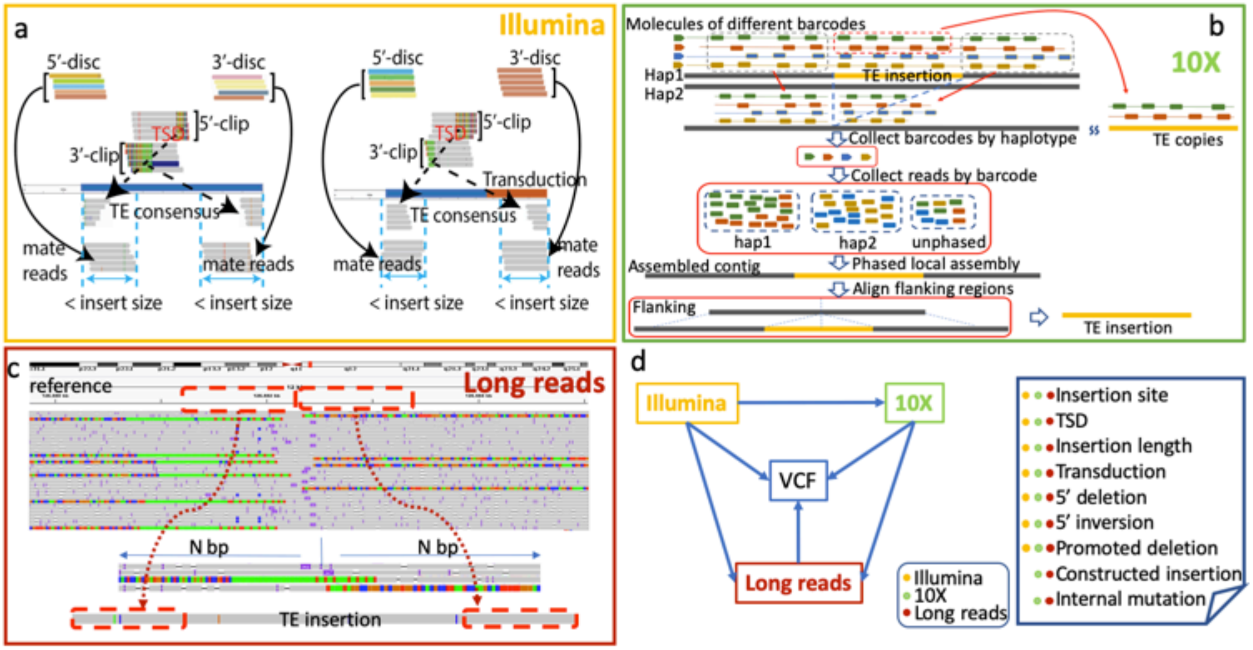

Fig. S1: A more detailed overview of TE insertion calling in xTea. **a,** Canonical TE insertion calling from paired-end short Illumina reads for TE insertion (left panel) and transduction (right panel). Candidate sites are collected from clipped reads, and those sites that do not have enough supporting discordant pairs are removed. Different from other tools, xTea also checks the pattern of the realigned clipped and discordant reads on the consensus (or copies). Both 5' and 3'-clipped reads form separate clusters on the consensus, and similarly for 5' and 3' discordant reads. The distance between the cluster formed from 5' (3')-clipped reads and the cluster formed from 3'(5')-discordant reads should be smaller than the library insert size (mean +/- 3 s.d.). For transduction of L1s and SVAs (right panel), most steps are the same, except that one side of the cluster is formed from realignments to the flanking regions of full-length copies rather than the consensus (or copies). **b,** TE insertion calling and phased assembly from 10X Linked-Reads. For each candidate site (detected as described in **(a)**), we first collect the barcodes from the anchor reads near the site. Because these anchor reads have already been phased, those collected barcodes are separated into three groups: haplotype 1, haplotype 2, and unphased. For each barcode in a group, we collect all the reads having the same barcode; thus, all collected reads are separated into three groups. Then we do local assembly for each group and align the flanking regions to the assembled contigs to call out the insertion sequence. **c,** TE insertion calling and assembly from long reads. First, clipped reads and insertion from the CIGAR field are collected to check whether a site has enough supporting reads. Then, for each candidate site, we assemble the collected reads and then align the flanking regions to the assembled contigs to identify the insertion. **d,** A hybrid calling scheme and the different output of each platform.

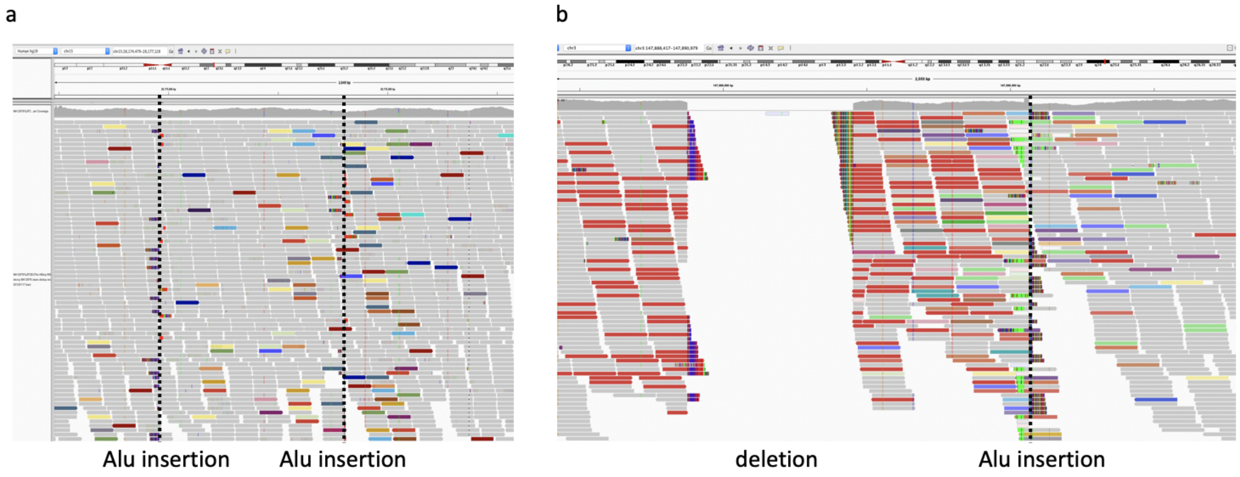

Fig. S2: Example IGV screenshots of TE insertions close to other SVs. a, Two adjacent Alu insertions result in discordant reads that are mingled together to obscure TE insertion calling. Some callers (e.g., MELT) fail to detect both of the Alu insertions. **b**, Discordant reads of an Alu insertion are mixed with a nearby deletion event. Some TE insertion callers (e.g., MELT) fail to detect such Alu insertions.

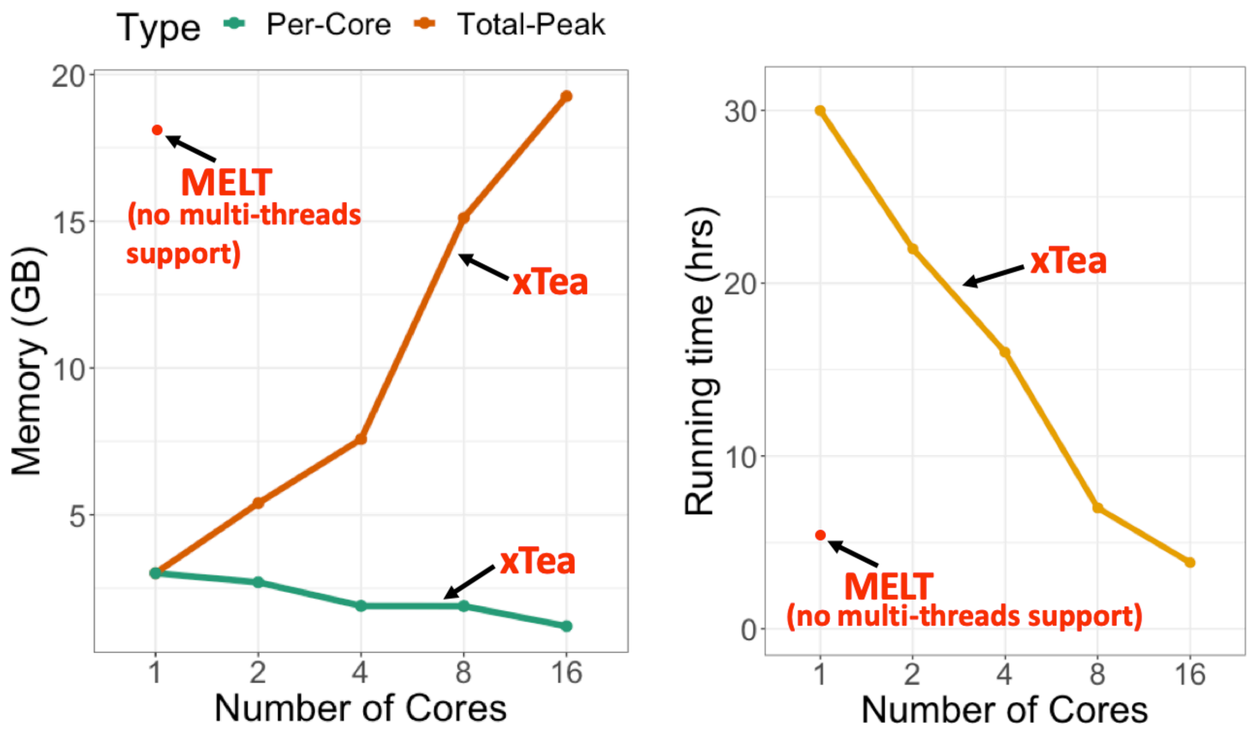

Fig. S3: Performance comparison between MELT and xTea on short reads. We benchmark the performance of MELT and xTea on HG002 (~45X coverage). MELT and xTea were run on Amazon Web Services (AWS) nodes with the following configuration: Intel(R) Xeon(R) CPU @ 2.30GHz, 16 cores and 32G memory. MELT, which does not support multiple threads/cores, took 5hrs and 13 mins. In comparison, xTea supports multiple threads/cores, and it took 6 hrs 55 mins and 3 hrs 50 mins when run with 8 cores and 16 cores, respectively. With xTea, the average amount of memory per core decreases with the increased number of cores.

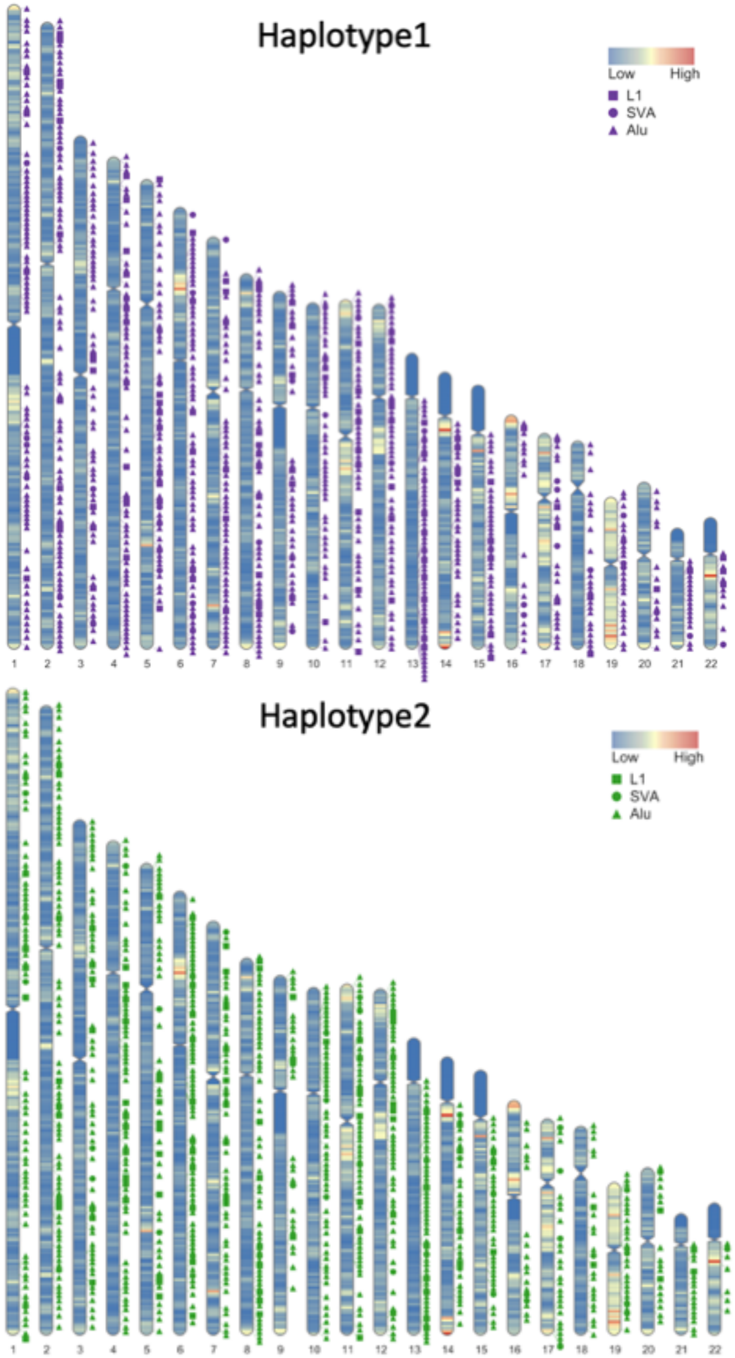

Fig. S4: Distribution of haplotype-resolved TE insertions for HG002. In total, 1,642 TE insertions (1,355 Alu, 197 LINE-1, and 90 SVA) were identified. HG002 is male and TE insertions from sex chromosomes cannot be not well phased, thus are not shown.

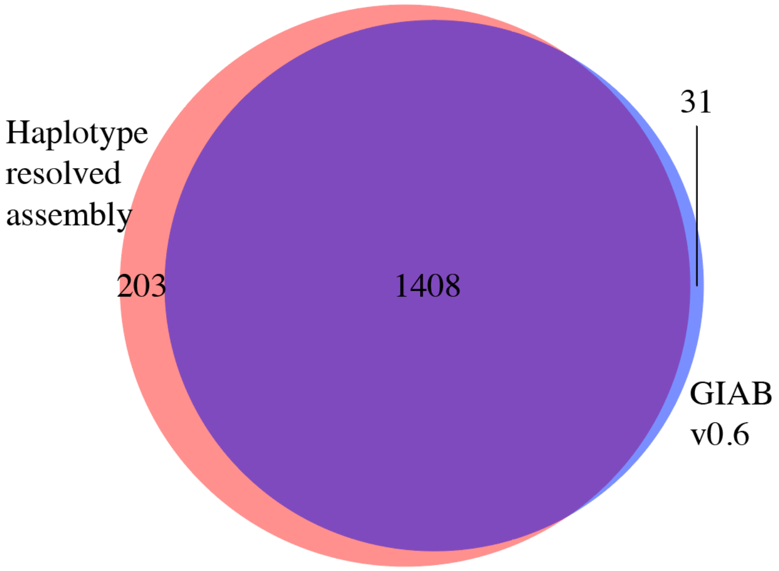

Fig. S5: Composition of the HG002 benchmark TE insertion set. The insertions were combined and annotated from two sources: GIAB v0.6 and haplotype-resolved assembly. We first collected all insertions >50bp from GIAB v0.6 and the haplotype-resolved assembly. To annotate the insertions, we ran RepeatMasker and selected any insertions that have at least some segment annotated as LINE-1, Alu, or SVA. Then, we manually inspected each annotated insertion using both IGV and RepeatMasker output, and selected 1,642 TE insertions as our final benchmark.

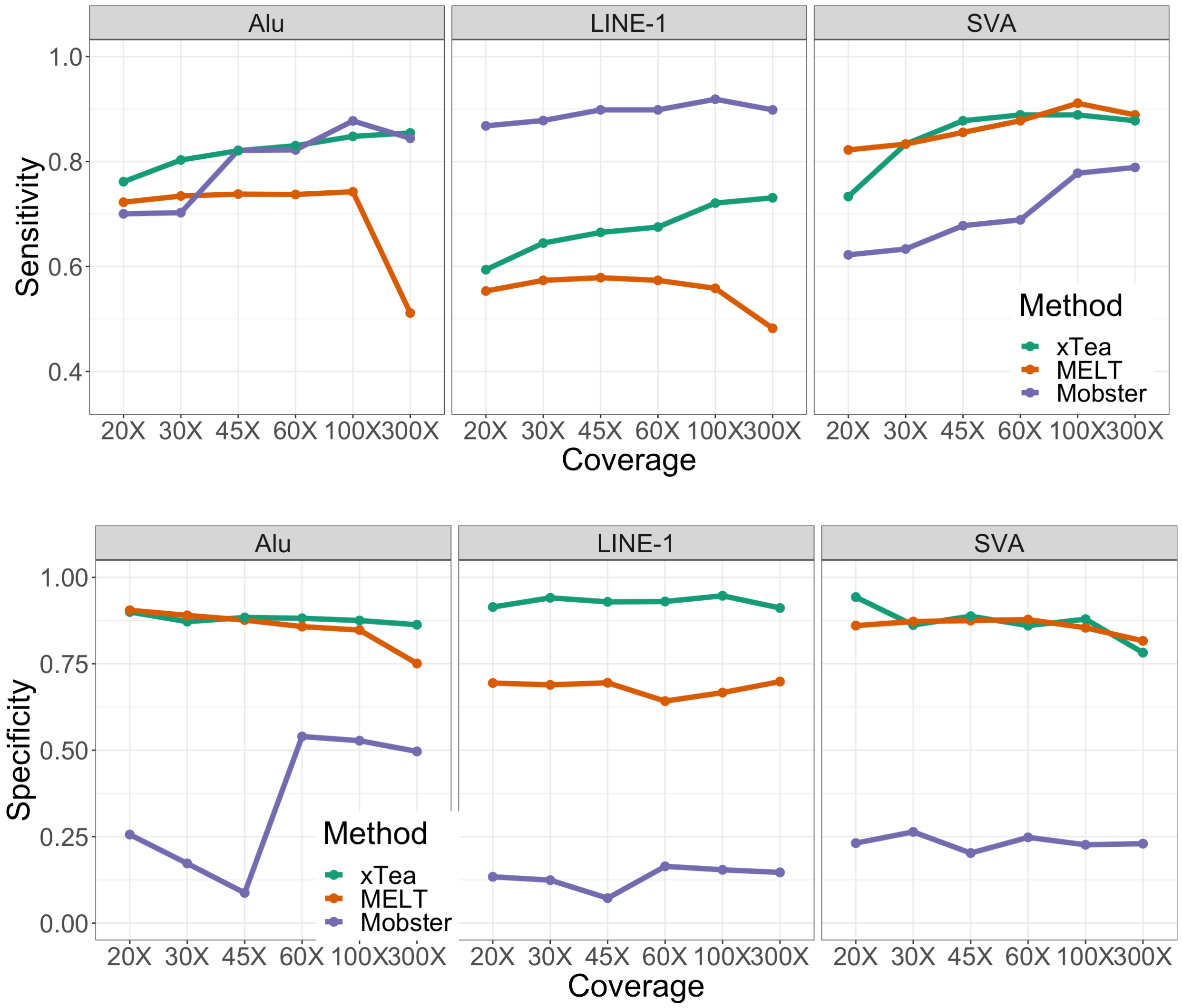

Fig. S6: Comparison of xTea, MELT, and Mobster in sensitivity and specificity on different coverages from short reads. xTea shows much better performance in both sensitivity and specificity than MELT for L1; its sensitivity for Alu is also better than that of MELT. Mobster shows higher sensitivity for L1 but much lower specificity for all three families compared to xTea and MELT.

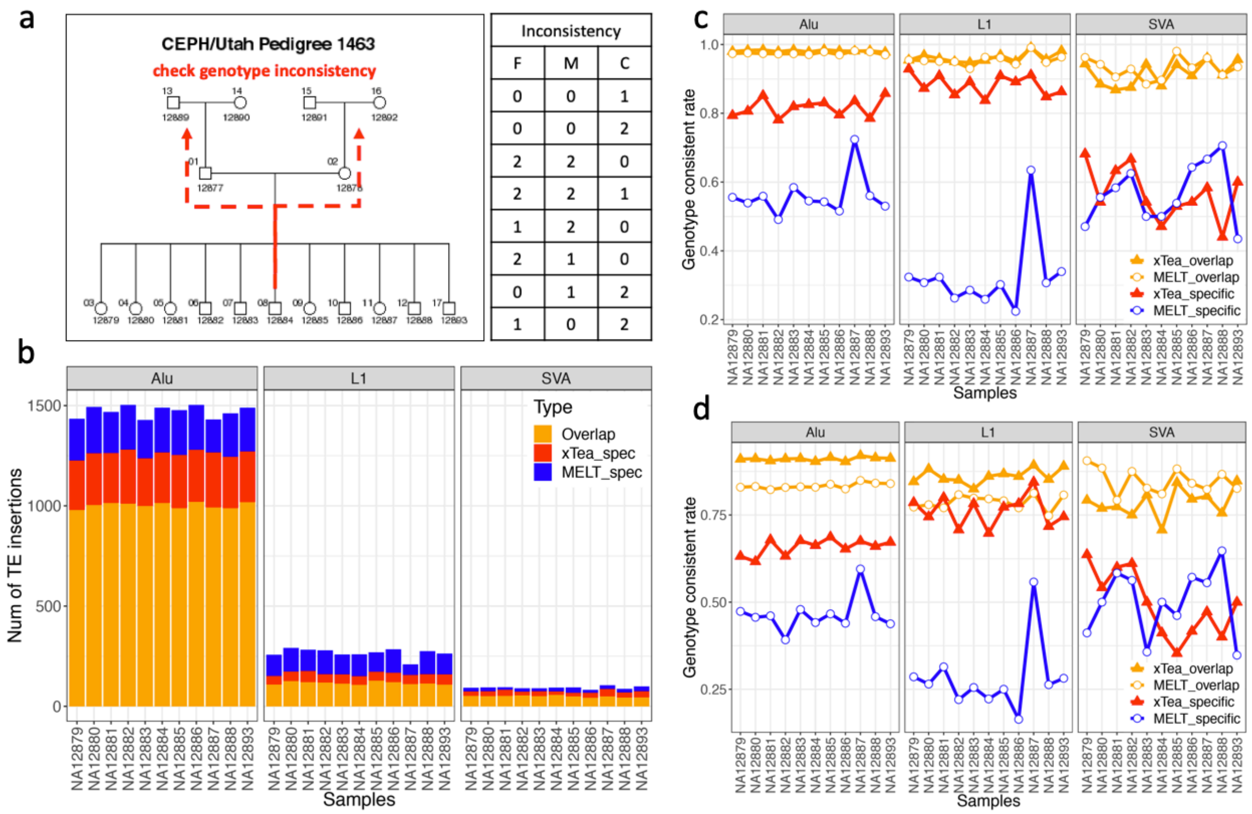

Fig. S7: Comparison of MELT and xTea on genotype calling with pedigree data from short reads. a, The left panel shows the relationship among the 17 members of the pedigree; the right panel shows the defined genotype inconsistency, where F, M, and C indicate the genotype of father, mother, and child respectively, and 0, 1, and 2 represent reference homozygous, heterozygous, and homozygous alternate, respectively. **b,** Number of overlapping and algorithm-specific Alu, L1, and SVA insertions for the 11 children. **c,** Genotype consistency between child and parents. Insertions shared between xTea and MELT show similar genotype consistency; xTea-specific insertions are much more consistent for Alu and L1 than MELT-specific ones. **d,** Genotype consistency for both child/parent and parent/grandparent. The overall consistent rate is lower than in **c**, but the trend is similar, with xTea performing much better than MELT for Alu and L1.

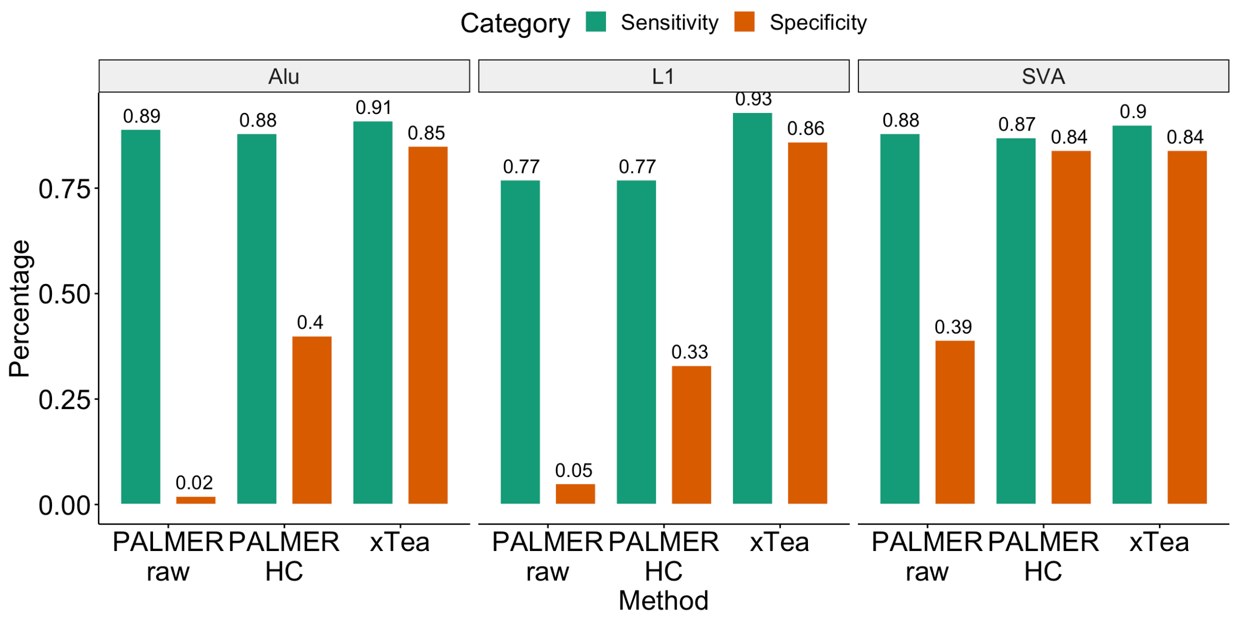

Fig. S8: Comparison between PALMER and xTea on HG002 HiFi long reads. "PALMER raw" is the initial output from PALMER, and "PALMER HC" is the high-confident set (filters applied). xTea outperforms PALMER in specificity, as PALMER reports a large number of false positives.

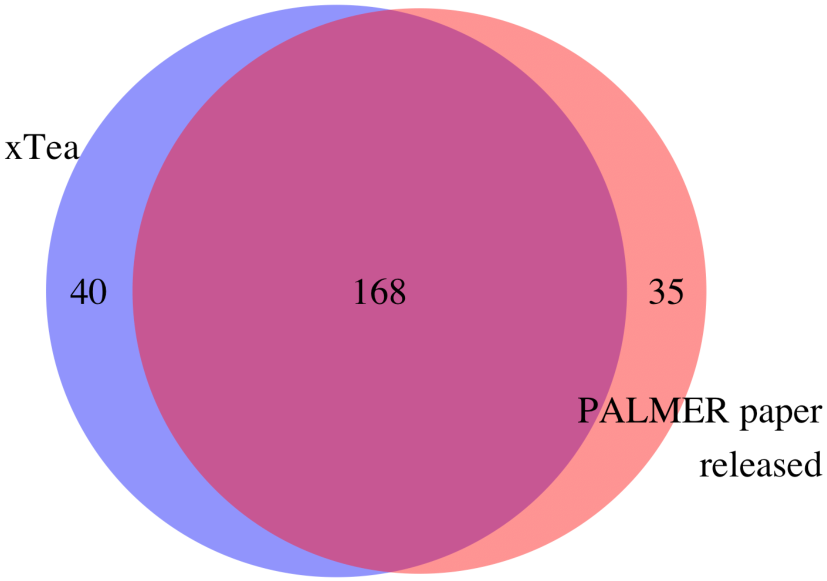

Fig. S9: Comparison between the L1 insertions called by xTea on NA12878 HiFi long reads and the call set released in the PALMER paper. Between the 208 insertions identified by xTea and the 203 manually-inspected insertions by PALMER, 168 were shared.

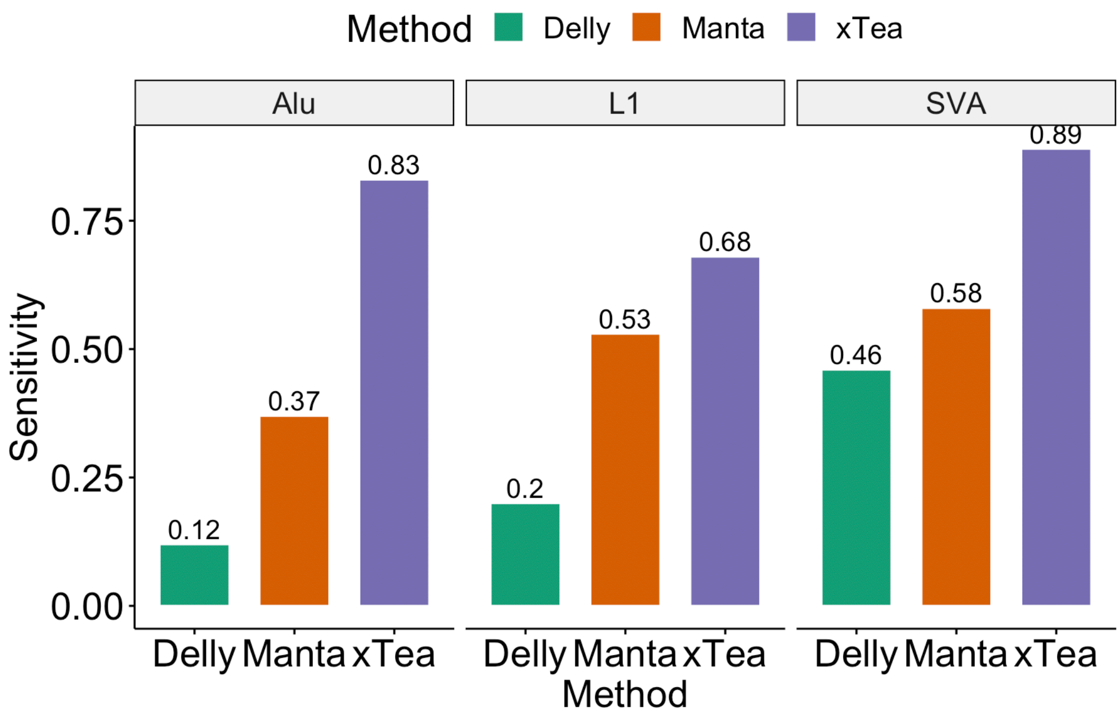

Fig. S10: Sensitivity comparison between Delly, Manta, and xTea on short reads. The algorithms were run on HG002 (~60X). xTea shows higher sensitivity for each TE family.

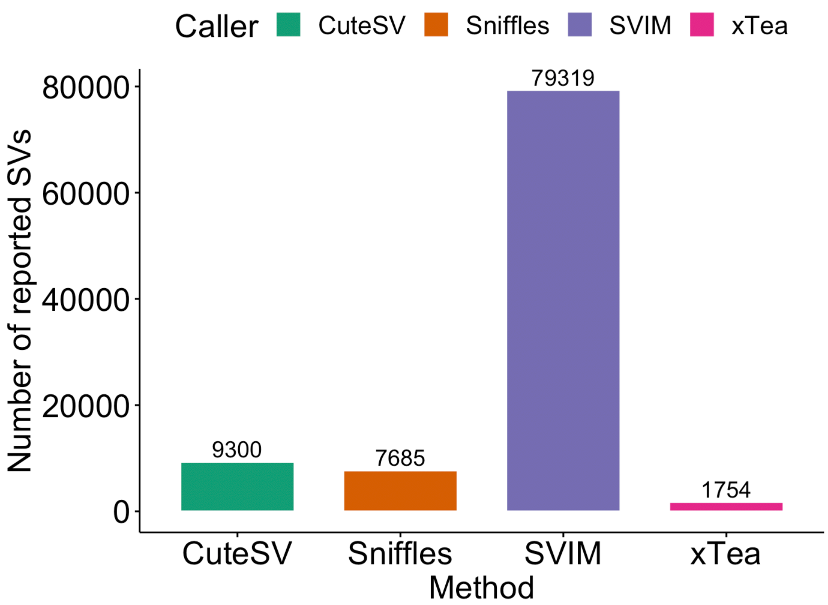

Fig. S11: Number of SVs reported on the HG002 HiFi long-read data. xTea is designed to detect TE insertions whereas the others are general-purpose SV callers. SVIM reports a large number of SVs, indicating a high rate of false positives.

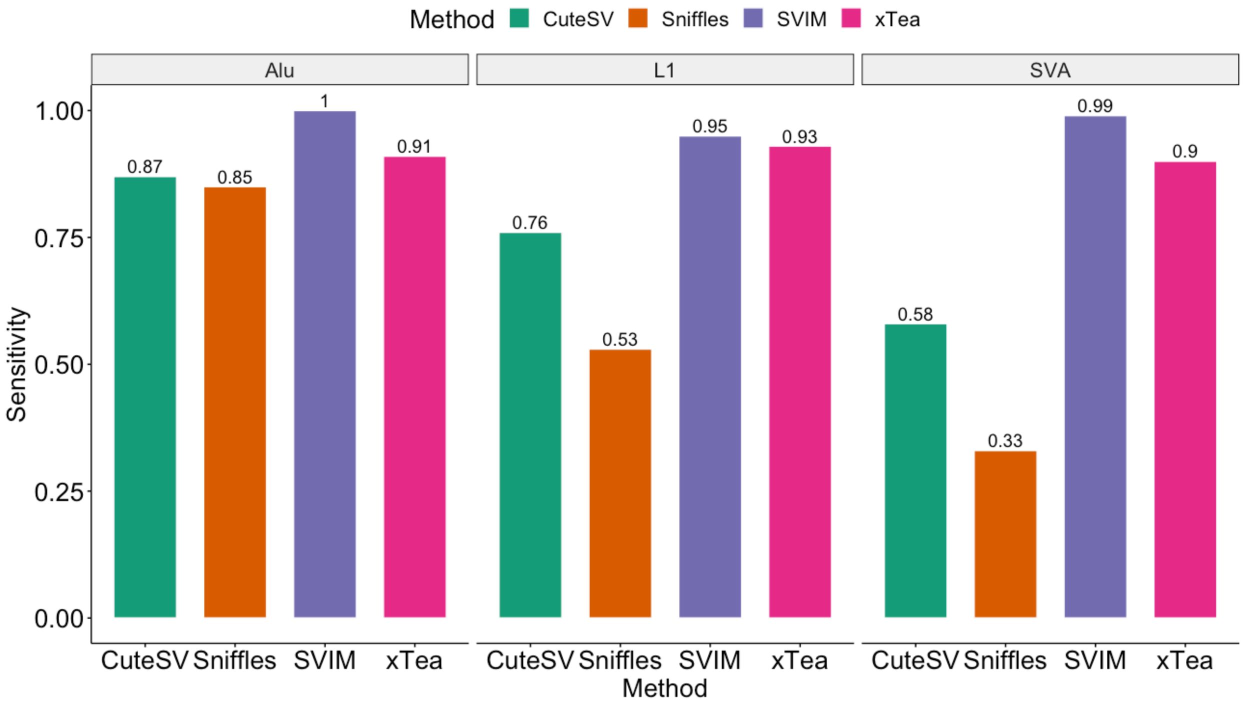

Fig. S12: Sensitivity comparison on the HG002 HiFi long-read data using the benchmark TE insertions. Sniffles and xTea construct the insertion sequences, whereas CuteSV and SVIM only report the breakpoints. Although Sniffles can construct the insertions, it shows low sensitivity for L1 and SVA. SVIM show highest sensitivity, but it reports lots of false positives (from Fig. S11).

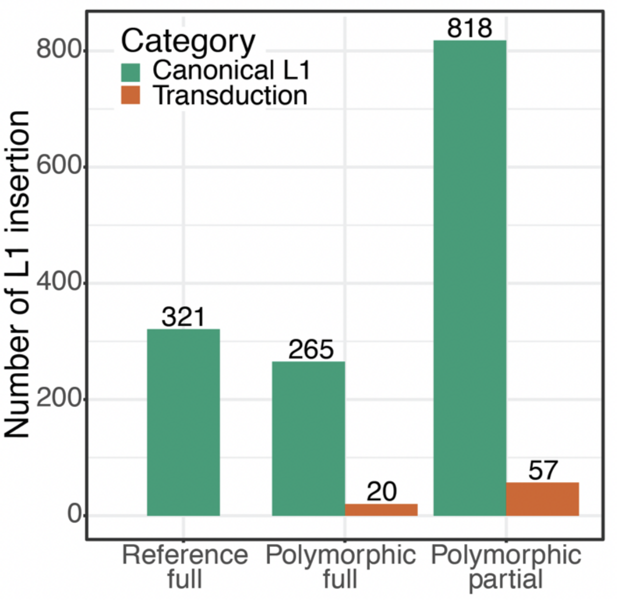

Fig. S13: Number of non-redundant polymorphic L1 insertions detected from the 20 long read samples. The reference genome (GRCh38) has 321 L1 full-length copies (defined as >6kb). From the twenty long-read samples, 285 polymorphic L1 insertions were detected, with 20 having 3' transduction. Also, 875 truncated polymorphic L1 insertions were detected, with source elements uniquely found for 57 transductions.

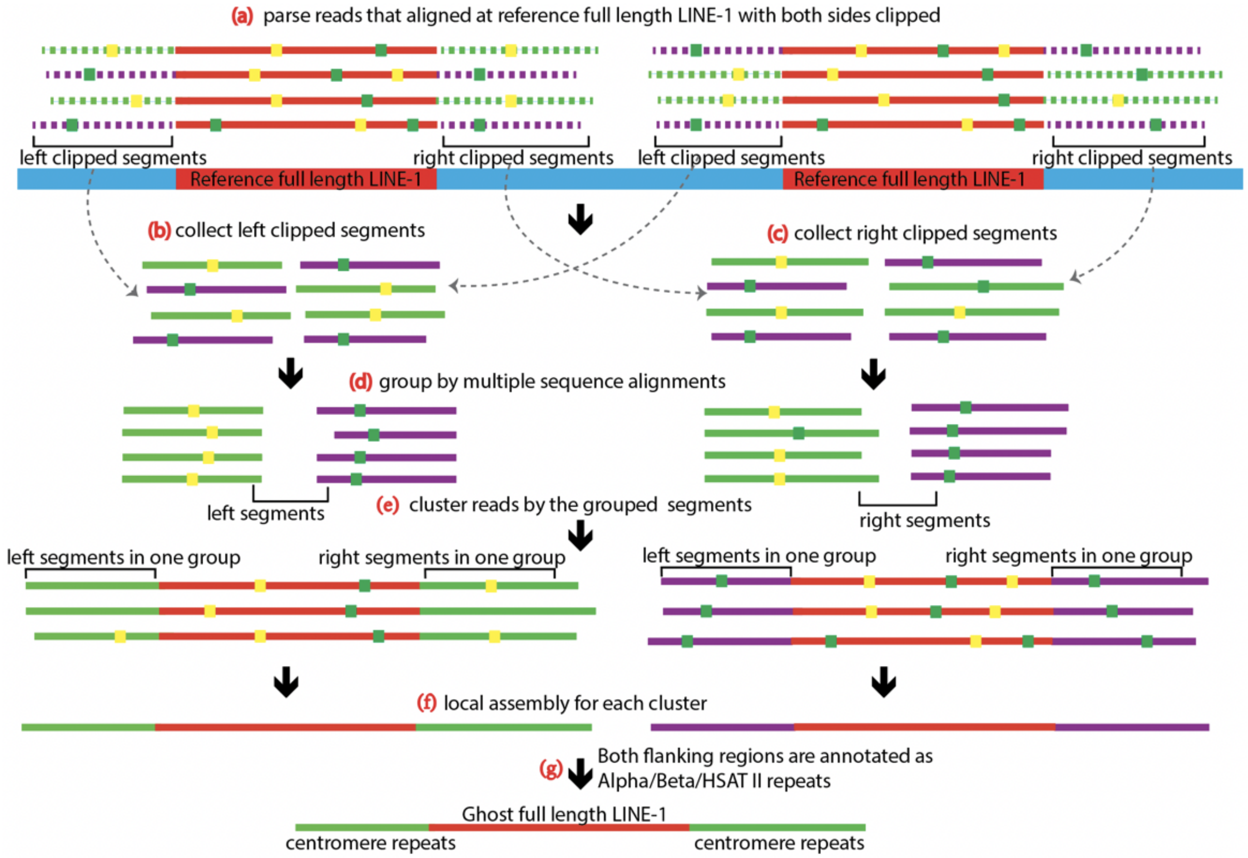

Fig. S14: "ghost" full length L1 identification from long reads. a, We collect all those reads that aligned to the reference full length L1 copies but with the two tail sides clipped. **b-c,**We collect the left and right clipped parts separately. We assume here the copies are in the same orientation; in practice, we adjust based on orientation. **d.** We do multiple sequence alignments, based on the results of which we group the segments. **e.** We select those reads (of a group) that have the left and right clipped parts each form one group. **f.** Then for each cluster of these reads, we do local assembly. **g.** We check the flanking regions of each assembled contig, and select assembled L1 copies that have both flanking regions masked as Alpha, Beta or HSATII repeats.

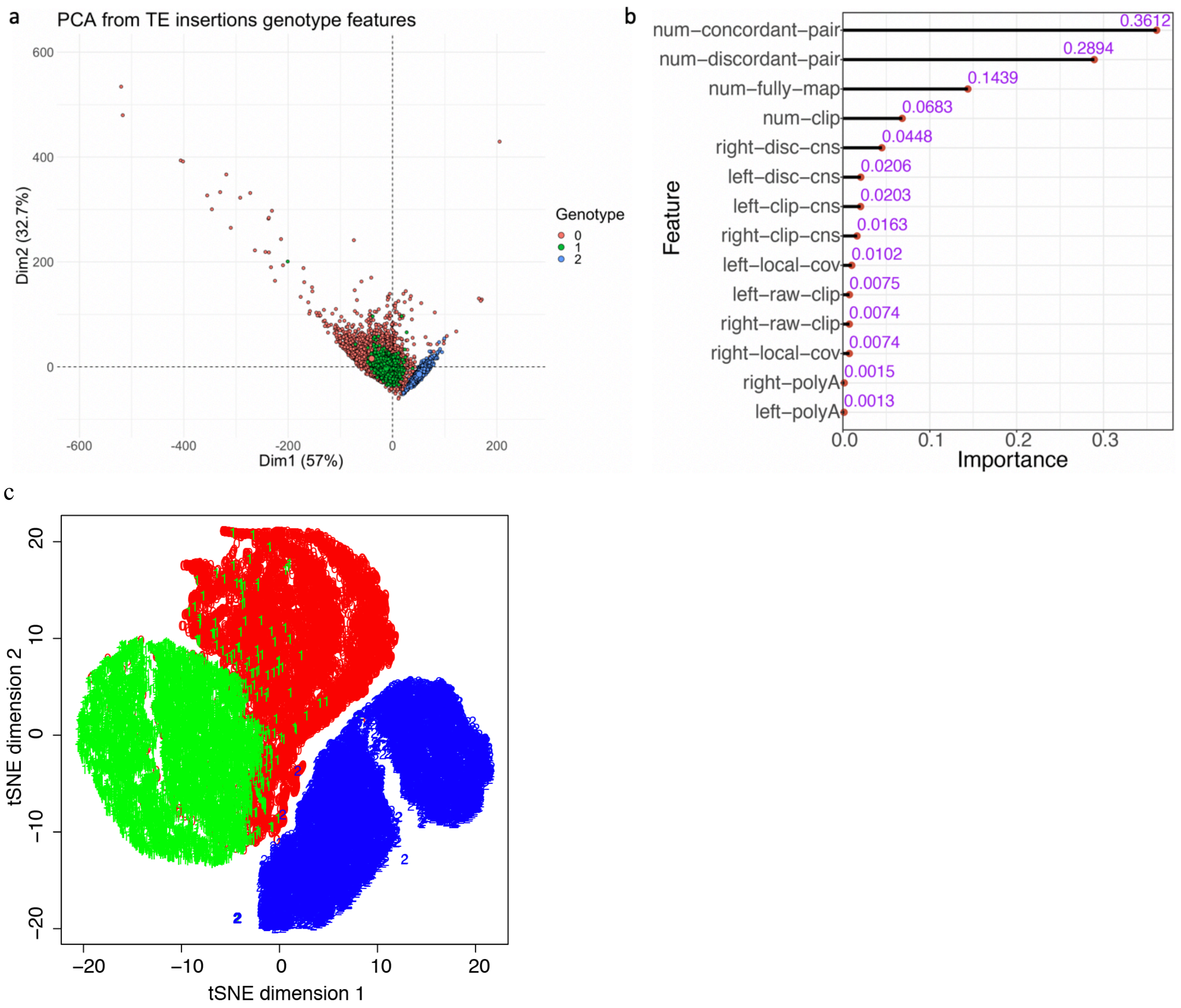

Fig. S15: Illustration of TE insertion genotype classification and feature importance. a, We randomly selected 3,000 homozygous reference (0, in red), 3,000 heterozygous (1, in green), and 3,000 homozygous alternate (2, in blue) sites from the training data, and ran PCA on them. Top two dimensions show that ~90% of the points can be clustered, which indicates that the training data are well classified. **b,** Feature importance evaluated from the random forest model training procedure. In total, 14 features are collected for genotype classification, among which "number of concordant pairs", "number of discordant pairs", "number of fully mapped reads at junction", and "number of clipped reads" are most important. **c,** tSNE clustering for the same 9,000 genotypes in figure a. Genotype 0, 1, and 2 are in red, green, and blue respectively. Note, we defined those false positive ones from the output of xTea as genotype 0 when we built the training set, which means they all at least have some "clip" and "discordant pair" support. Thus, for some cases, some features are shared between 0 and 1 in our training data, which is why some are not well "classified" from both PCA and tSNE view.

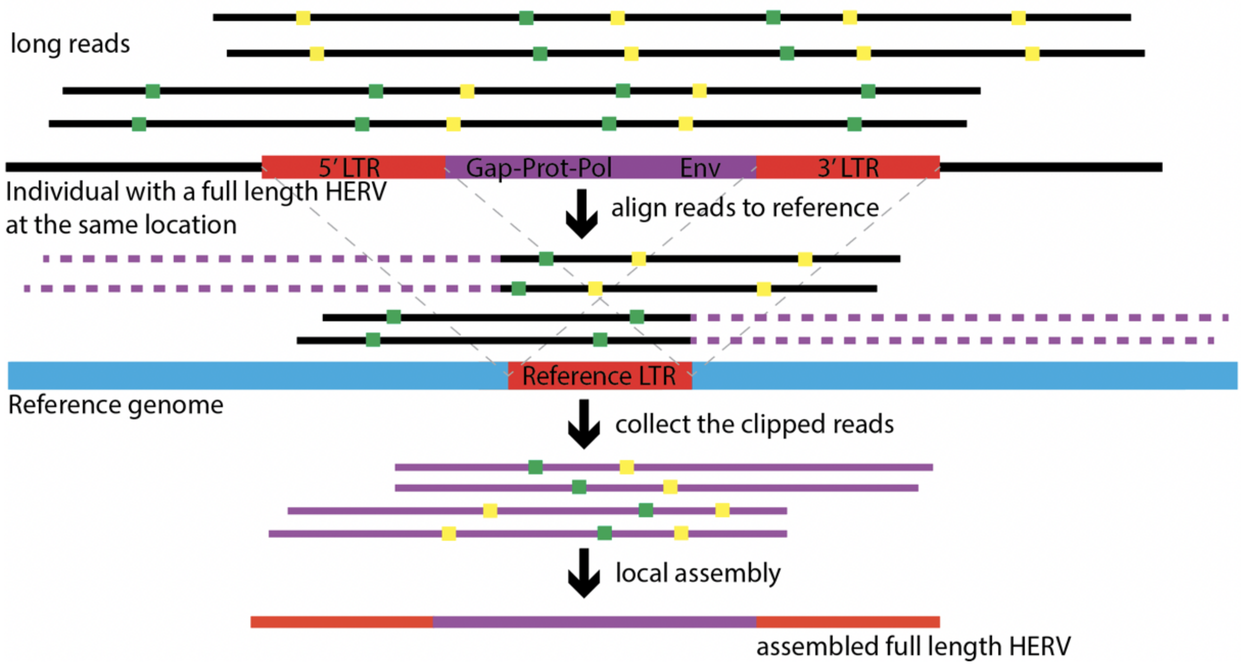

Fig. S16: Dimorphic HERV calling from long reads. xTea first checks each reference-annotated LTR to determine whether there are reads clipped at the breakpoints. Specifically, reads left clipped at LTR start position and right clipped at LTR end position. Then, for each group of collected reads, xTea performs local assembly to construct the full copy. In addition, xTea aligns the LTR back to the assembled contig to annotate the two side LTRs and the internal provirus.

Method	Coverage	F1	Sensitivity	FDR	TP	FP	FN
Illumina	60X	0.855	0.830	0.118	1125	151	230
Illumina+10X	120X	0.832	0.850	0.185	1152	262	203
10X	60X	0.767	0.821	0.281	1113	434	242
Nanopore	45X	0.875	0.974	0.206	1320	342	35
PacBio CLR	45X	0.875	0.959	0.196	1299	316	56
PacBio HiFi	30X	0.876	0.906	0.151	1227	219	128

Tab. S1: Performance of xTea in detecting Alu insertions on different sequencing platforms on sample HG002.

Method	Coverage	F1	Sensitivity	FDR	TP	FP	FN
Illumina	60X	0.782	0.675	0.070	133	10	64
Illumina+10X	120X	0.790	0.695	0.0867	137	13	60
10X	60X	0.743	0.645	0.124	127	18	70
Nanopore	45X	0.8280	0.868	0.208	171	45	26
PacBio CLR	45X	0.831	0.848	0.185	167	38	30
PacBio HiFi	30X	0.895	0.929	0.137	183	29	14

Tab. S2: Performance of xTea in detecting L1 insertions on different sequencing platforms on sample HG002.

Method	Coverage	F1	Sensitivity	FDR	TP	FP	FN
Illumina	60X	0.874	0.889	0.140	80	13	10
Illumina-10X	120X	0.838	0.922	0.231	83	25	7
10X	60X	0.806	0.833	0.219	75	21	15
Nanopore	45X	0.742	0.733	0.250	66	22	24
PacBio CLR	45X	0.753	0.778	0.271	70	26	20
PacBio HiFi	30X	0.871	0.900	0.156	81	15	9

Tab. S3: Performance of xTea in detecting SVA insertions on different sequencing platforms on sample HG002.

Genotype		PCR		
		0/0	0/1	1/1
**xTea predicted**	0/0	659	0	0
	0/1	0	126	11
	1/1	0	0	32

Tab. S4: Comparison of xTea with a PCR benchmark data (Payer et al. 2017) in genotype calling. The PCR dataset reported validation results at 145 Alu sites for 90 samples from the 1000 Genomes project including 45 high-coverage (~30X) samples. We downloaded these 45 samples from the 1000 Genomes Project data portal (https://www.internationalgenome.org/data-portal/data-collection/30x-grch38) and ran xTea on them to call and genotype TE insertions. We converted the coordinate of the PCR benchmarked insertions (Alu only) from hg18 to hg38. 19 sites are overlapping between xTea calls and the PCR benchmark and have PCR-genotype as "0/1" or "1/1" in at least one sample. 27 PCR genotypes do not have any information in their released table, and are removed. Thus, in total 19*45-27=828 genotypes (659 "0/0", 126 "0/1", and 43 "1/1") are obtained for comparison. For xTea, if no insertion is reported for the given site, then the genotype of this site for this sample is "0/0". The results show highly consistent genotypes between xTea and the PCR benchmark data, except for the 11 sites that xTea genotyped as heterozygous (0/1) but the PCR data showed homozygous (1/1). These 11 genotypes may have been incorrectly predicted by xTea, but it is also possible that the genotypes from PCR are imprecise.

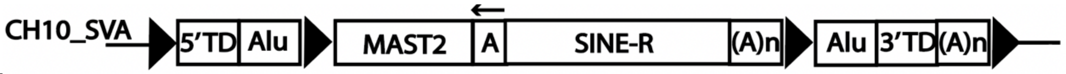

Label	Start	End	Orientation	Sub-family	Family
5' TD	23	110	C	MIR	SINE/MIR
	185	321	+	AluSc	SINE/Alu
Fusion	533	575	+	(GCC)n	Simple_repeat
	697	1459	+	SVA_F	Retroposon/SVA
SVA	1086	1902	+	SVA_F	Retroposon/SVA
	1523	2692	+	SVA_F	Retroposon/SVA
	2717	3016	+	AluSp	SINE/Alu
Fusion	3097	3117	+	(A)n	Simple_repeat
	3349	3589	+	L1ME3G	LINE/L1
3' TD	3590	3682	+	L1MA10	LINE/L1
	3683	3761	+	L1ME3G	LINE/L1
PolyA	3774	3794	+	(A)n	Simple_repeat

Tab. S5: RepeatMasker output of one CH10_SVA copy. Here, we show a more complex example of an SVA insertion, called CH10_SVA, that was fused with the MAST2 gene and is still actively creating new insertions in the human genome. In the table, we show the information from RepeatMasker output (except for the column "Label"). This single SVA insertion was annotated as 8 subfamilies, thus we cannot tell the family of this insertion only by RepeatMasker annotations. In addition, RepeatMasker cannot properly annotate insertions with transductions, which comprise >15% of L1 and >15% of SVA insertions. Because the transduction sequences can be masked to one or more repeat segments of any TE type or TE subfamilies. With these non-unique family annotations from RepeatMasker, we cannot tell which "family" these insertions are. In contrast, xTea has a stand-alone module to annotate TE insertions into detail, such as TE subfamily, target site duplication (TSD), polyA tails, internal SVs and transductions.

Sample ID	Sex	Super-family	Technology
HG03098	Male	AFR	Nanopore
HG02055	Male	AFR	Nanopore
HG01243	Male	AFR	Nanopore
HG03492	Male	SAS	Nanopore
HG02723	Female	AFR	Nanopore
HG02080	Female	EAS	Nanopore
GM24143	Female	CEU	Nanopore
NA19240	Female	AFR	PacBio CLR
HG002	Male	CEU	PacBio HiFi
HG00733	Female	AFR	PacBio CLR
HG01352	Female	AMR	PacBio CLR
NA12878	Female	CEU	PacBio CLR
HG00268	Female	CEU	PacBio CLR
HX1	unknown	EAS	PacBio CLR
NA19434	Female	AFR	PacBio CLR
AK1	unknown	EAS	PacBio CLR
HG02059	Female	EAS	PacBio CLR
HG02106	Female	AMR	PacBio CLR
HG00514	Female	EAS	PacBio CLR
HG04217	Female	SAS	PacBio CLR
HG02818	Female	AFR	PacBio CLR

Tab. S6: Long read samples used in the study. For each sample, we show the sex, population super-family and from which platform the sample is sequenced. In all, 7 samples are sequenced with Oxford Nanopore and the rest 13 samples are sequenced with PacBio platform (one is sequenced with HiFi).

Full length L1 ⇨ (row 2553851)

Start	End	Orientation	Sub-family	Family	cns_start	cns_end
2	900222	C	ALR/Alpha	Satellite/centr	878975	1
900250	1040062	C	ALR/Alpha	Satellite/centr	137408	1
1040090	1085285	C	ALR/Alpha	Satellite/centr	45042	1
1085313	2553843	C	ALR/Alpha	Satellite/centr	1434461	1
2553851	2559901	+	L1HS	LINE/L1	124	6155
2559902	2673272	C	ALR/Alpha	Satellite/centr	111965	1
2673349	2673449	C	ALR/Alpha	Satellite/centr	164	59
2673482	2673599	C	ALR/Alpha	Satellite/centr	124	1
2673771	2673877	C	ALR/Alpha	Satellite/centr	120	8
2674112	2674211	C	ALR/Alpha	Satellite/centr	169	60
2674844	2674901	+	(TTTTCTA)n	Simple_repeat	1	58
2675395	2675445	C	ALR/Alpha	Satellite/centr	58	8
2676472	2678222	C	ALR/Alpha	Satellite/centr	1760	1
2678244	2678511	C	ALR/Alpha	Satellite/centr	264	1
2678531	2681227	C	ALR/Alpha	Satellite/centr	2738	1
2681417	2681548	C	ALR/Alpha	Satellite/centr	140	2
2681759	2681953	C	AluYc3	SINE/Alu	285	92
2682269	2682344	C	ALR/Alpha	Satellite/centr	111	35
2682347	2682404	+	(TTGTTT)n	Simple_repeat	1	56
2682546	2682710	C	ALR/Alpha	Satellite/centr	170	1
2683600	2683707	C	ALR/Alpha	Satellite/centr	110	1
2683795	2684059	C	ALR/Alpha	Satellite/centr	272	1
2684222	2684267	+	(ATTTT)n	Simple_repeat	1	45
2685944	2686531	C	L1P3	LINE/L1	4339	3742
2686976	2687010	+	(TTCTT)n	Simple_repeat	1	35
2687065	2837682	C	ALR/Alpha	Satellite/centr	147952	1

Tab. S7: chrX centromere full length L1 of CHM13. An example centromere full length L1 is annotated in chrX centromere, which is fully assembled with Oxford nanopore long reads, HiC and sequencing data of several other platforms. The insertion has a minor deletion at the 5' side, and of almost full length with the flanking regions are annotated as Alpha satellite repeats.

LINE-1 sub-family	Number of copies
L1PA5	3
L1PA4	17
L1HS	12
L1PA2	29
L1PA3	53

Tab. S8: Potential centromere full length L1s from CHM13 telomere to telomere assembly. We run RepeatMasker on the CHM13 v1.0 assembly (Miga et al. 2020) and select all the full length (>5950bp) L1s with at least one side flanked with satellite repeats (>5000bp) and found 114 potential centromeric full length L1s, out of which 12 are L1HS. Even with long reads, different centromeric L1s cannot be distinguished from each other when they have the same long centromeric flanking repeats. Due to this limitation, the ghost L1 detection module in xTea can call full length L1 copies whose flanking sequences could be distinguished. Thus, we checked how many L1s out of 114 copies from the assembly are unique in terms of their flanking sequences by grouping them according to their flanking sequences. We obtained 19 unique copies because 96 copies carried the same flanking repeat sequences.
