# Supplementary material for: Comprehensive identification of transposable element insertions using multiple sequencing technologies
Source: Nat Commun. 2021 Jun 22;12:3836. doi: 10.1038/s41467-021-24041-8 (PMC8219666; doi:10.1038/s41467-021-24041-8)
Supplement: Supplementary file 2 — Peer Review [file 41467_2021_24041_MOESM2_ESM.pdf]

REVIEWER COMMENTS

Reviewer #1 (Remarks to the Author):

In this paper, Chong Chu et al nicely describe a method for detecting mobile elements of various types from both long and short read sequencing data. This method, xTea, is a helpful addition to the array of techniques currently available to call these events in both short and long read data. The biggest improvements here are the application to both long and short read data, the clear annotation of even LTR elements, and the annotation of the elements that are called. The authors use their method to call insertions in an extensively characterized dataset, HG002, and generated haplotype resolved calls in this genome. They find great improvements in accuracy and precision, especially for L1s, with long read data, and their method performs quite well. This is especially true, given the nature of long reads, for L1s in repeat regions, such as the centromere and those that accompany structural rearrangements. The methods and entire paper are well written. A previously developed tool
does have the benefit of using long read data, and PALMER comparisons would actually likely benefit the authors manuscript.

The major critiques I have of this paper are the following;
1) The authors mention PALMER, which calls L1s in long read data, but do not compare their method to this existing tool. Additionally, they only utilize HiFi reads; it would be nice to see how their cigar/split read approach performed with less accurate CLR reads. Furthermore, the comparison to PALMER, if the authors improve their user friendliness, would be astounding. The multithreading is already an improvement. A bioconda package would be a larger improvement. The ability of the method to be adapted to novel genomes other than human given a library of mobile element consensus sequences could be an important improvement to the existing technique.
2) The confusion matrix for HG002 calls could use some filling in. I would appreciate knowing the # of false positive calls from xTea.
3) The Docker platform is nice to have, but their script on github needs work. A bioconda package would make the tool more widely use-able, given the dependencies for the code. Additionally, the README file does not currently contain all of the packages/versions and parameters required to run the tool, and does not state whether the tool can perform in python 3. Further issues include hardcoded email addresses in the shell script that are particular for running the job on slurm. The script does seem to be scheduler dependent, and that is a large issue for submitting the jobs on another HPC that uses Torque/MOAB.
4) The long read work flow had missing files that required import from the short read version (rep_lib_annotation.tar.gz). Additionally, there are many parameters that are not explained, and need adequate commenting and sections in the README or help pages.
5) The comparison between long read and short read data are a bit unfair; the genotype data in MELT are not robust, and TypeTE is far improved. The use of insertion calls from Long read callers like PBSV or SVIM to overlap with their tool would give a better picture of the unique knowledge gained by using xTea over long read SV caller annotation, given that short read data

are notoriously bad for identification of insertions. A comparison with PALMER would be even more useful.

Reviewer #2 (Remarks to the Author):

Transposable elements (TEs) frequently occurs in the human genome, and TE insertions are involved in human diseases when the insertion disrupt human genes. The authors developed a novel computational tool (xTea) for identifying transposable element insertions from short-read, linked-read and PacBio long-read whole-genome sequencing data. The main advantage over existing approaches is that xTea can be applied to long-read data, which is expected to have highly sensitivity and specificity to detect TEs, compared to conventional Illumina short-read sequencing. They also created a high-confidence set of TE insertions for the GIAB HG002 genome. The authors benchmarked xTea on this data set and a large pedigree data set. The authors showed that xTea outperformed MELT and TraFiC-mem.

xTea identifies TE insertions using methods that is also used by general insertion callers (e.g. discordant read pairs, clipped/split reads, insertion within read alignment, local assembly), with some optimizations. The authors compared xTea to some other TE insertion callers. However, it would be interesting to compare xTea with a general-purpose insertion detection pipeline. For example, a general-purpose SV detection pipeline can use Manta/Pindel/fermikit to call insertions for short reads and SMRT-SV/PhasedSV/Sniffles to call insertions for PacBio long reads, and use Repeatmasker to annotate the repeats. The reason why it is important is that as a user of long-read sequencing data, I will never use an existing TE caller (as they are not really designed for PacBio/Nanopore data) for TE calling. There are a number of SV callers that are published in the past a few years, each claiming superior performance, and most (if not all) of them allows the identification of TE insertions
(it require slight post-processing of results by annotation by repeatmasker). A comparison to these tools on long-read data would be more informative, even though these tools were general purpose SV calling tools.

The paper showed benchmarks for PacBio HiFi reads, but claimed that xTea also works for regular long-read sequencing platforms (e.g. PacBio raw reads and Oxford Nanopore long reads). Intuitively the main difference would be that regular long-read data have higher basecalling error rates, but at the same time can offer longer read length to enable more accurate alignment and SV calling. Since all the “benchmark genomes” are now extensively sequenced by multiple technologies, it would be important to benchmark these genomes using conventional PacBio data as well as Nanopore data, so that readers have a good sense of the

performance of the algorithms under different scenarios. These data (for example HG002 genome) are high-coverage public datasets on multiple technical platforms.

For similar reasons, it would be interesting to see if you can validate the centromeric L1 using data from other platforms (e.g. ultra-long nanopore reads, which is available for HG002). The ability to computationally validate specific predictions using a different (somewhat orthogonal) technical platform would yield confidence on the predictions on a challenging call in an extremely challenging area of the genome.

Additionally, GIAB themselves also released their own benchmark SV set on HG002 ("The final benchmark set contains 12745 isolated, sequence-resolved insertion and deletion calls =50 base pairs (bp) discovered by at least 2 technologies or 5 callsets"). It would not be difficult to simply annotate their calls and compare to your calls. Note that in their Figure 3 of GIAB, they already showed the expected peaks for Alu insertions and deletions near 300 bp and for full-length LINE1 insertions and deletions near 6000 bp. I am also a bit confused when reading this paper, since it seems that the authors somehow used the GIAB calls (~9.9K calls in a specific version of release plus assembly-based calls) to build their benchmarking data set, rather than using the xTea to help build a better benchmarking set. Nevertheless, if the authors want to claim that their "1,642 haplotype-resolved high-confidence TE insertions (1,355 Alu, 197 L1 150 and 90 SVA insertions; Fig. S5)" is of
better quality than what the GIAB has released to the public domain, some evidence needs to be shown and some comparisons need to be done. I also have the same concern of not using Nanopore data here in xTea analysis (they presented Illumina, 10X and HiFi data); note that the GIAB benchmarking set was built using 5 callsets.

In Figure 2, I do not understand the rationale of combining Illumina and 10X Genomics Illumina data together and present the results. In principle, if xTea can combine results from multiple technologies and generate consensus calls, it would be ideal to add a real long-read technology based caller in generating such results. This is important because HiFi alone actually achieves better performance than the "Illumina-10X" combination in Figure 2b.

In addition to HG002, the authors should also create the same benchmarked xTea data sets on other GIAB genomes, such as HG001, and the parents of HG002. All these genomes are extensively sequenced by multiple sequencing technologies, including Illumina sequencing and PacBio sequencing and Nanopore sequencing, and the data are publicly available. Part of the reason why people use HG002 nowadays is the availability of parental information, so such analysis should yield additional insights into the performance of any software tools to call mutations, as well as the intrinsic properties of specific type of mutations in terms of de novo mutations rates and stability in specific genomic regions.

I think the ability to find somatic L1 insertion is an interesting topic to address, given the known role of re-activated L1 insertion in tumor progression. However, the current paper used only one single lung cancer sample, and compared to a different method called TraFiC-mem here. I am not sure what is the purpose here exactly and why this particular sample is used (it does not look like it is a well known benchmarking sample with a set of known somatic TE, and MELT is not used here either). Given that a number of cancer cell lines with matched germline cells are sequenced by PacBio or Nanopore (in addition to Illumina), I would think it makes more sense to perform comparison on something where multiple orthogonal technologies have been used to generate data, to evaluate performance of the method on somatic mutations. The ability to generate more calls in a Venn diagram does not necessarily translate to improved performance of xTea over TraFiC-mem.

Around Page 17 line 320, the authors described their efforts in finding novel HERV. I am not familiar with this field but I found it to be interesting. The Figure 5c and 5d are not particularly informative and I cannot tell what information it intends to present: among the six known HERV and six novel ones, I do not see which is which, and the text does not mention it either. I feel that this entire section can benefit from some additional background introduction to HERV, so that readers have a better understanding of current knowledge on them, and explain how/why current Nanopore-based genome assembly cannot be used to examine and identify more HERVs (from what I read, if there are de novo genome assembly then it is straightforward to directly call HERV from the assembly itself).

I do not really see what is the “Machine learning-based TE insertion genotyping for short reads”. No description was given in the paper, in either Methods or Results section (except an illustrative panel in Figure 1), except that 14 features are used for each candidate. As a methods paper, details on the actual methods are needed. The reported 99.7% accuracy is also somewhat concerning as it is a bit too high, yet an examination of the statement shows that they have >1.3 million TE sites yet only 19K non-TE sites in the analysis which is a highly unbalanced set.

Some additional minor issues are noted below:

In page 4, line 57. This is wrong. The long reads can be much longer than 15 kb. In fact, in most Nanopore sequencing data that we use today, even the N50 length itself is already a lot higher than 20kb.

In page4, line 59. “To date, PALMER21 is the only tool designed for long reads, but it is limited to detection of canonical L1 but not Alu or SVA insertions.” This is not a fair statement. Almost all long-read SV callers can detect TE (users have to annotate the SV calls to know if they are L1 or Alu), but they are general purpose callers, not something specifically designed for TE calling.

In page 12, figure 3a. It is better to show both recall, precision.

In page 22, line 435. The author removed genomic regions with extremely high molecule coverage (> 250X) in the linked-read sequencing data. However, 250X is not extremely high for molecule coverage (I think you mean the coverage of reconstructed molecules/fragments from linked reads). In a typical 30X linked-read sequencing data, the average molecule coverage is about 150X.

In line 366. The polymorphic TE insertions are not necessarily more recent the TEs in the reference genomes.

In figure S6, the points with genotype "0" and "1" are not well separated. I didn't see "~90% of the points can be clustered". It would be better if you try more methods, such as tSNE.

General comments: since the method can be applied to both short and long read data, it is best to indicate in the legend of each figure what data sets are used for the results. This helps readers understand the results better, as some figures are purely based on short-read sequencing.

Reviewer #3 (Remarks to the Author):

I have now reviewed the manuscript of Peter J. Park and co-authors entitled "Comprehensive identification of transposable element insertions using multiple sequencing technologies". The authors present a new version their bioinformatic tool TEA, xTEA. xTEA detects and genotypes non-reference human transposable element insertions. xTEA allows users to use genomics data from a wide range of sequencing technologies including short, short-tagged and long read sequencing.

The paper has a strong focus on human health and the potential of their method for gene therapy.

The method is original and provide a significant enhancement of the currently published alternatives (see 'Strengths'). In my opinion, the methodology is accurate and incorporate state-of-the-art tools and algorithms. As of its suitability for publication in Nature Communications, I will likely support this decision if the authors responds to the points listed in 'Weaknesses'. I also include a list of questions or remarks that I will be grateful for the authors to reply, even though these issues do not dispute the reliability of the method (see 'Comments/Questions').

'Strenghs'

I my opinion, xTEA provide a significant enhancement of the current methods available for human TE data analysis. In particular:

- Ability to use and combine short, long and synthetic long reads (Ill. 10X) of the most popular sequencing technologies.

- Significantly higher sensitivity and genotyping accuracy for L1 elements which are historically hard to detect and genotype accurately.

- Implementation of a new, original genotyping algorithm for TE based on machine learning

- Higher or equivalent overall performances with most likely competitor method (MELT 'discovery')

- Detection of ERVs, and genotyping of full-proviral vs solo-ltr HERV insertions.

- Detection and classification of TE-related structural variants

- New high-quality TE genotype reference dataset.

'Weaknesses'

- My principal critique concerns the current availability of user support. Although the software and basic instructions are available online (via GitHub), the current manual is in my opinion too short and does not allow a new user to reproduce seamlessly the experiments shown in the paper. While the depiction of the pipeline is very clear in the manuscript, the instructions online did not allow me to perform a complete analysis and combine the different modules at the time of the review. Though this is a time-consuming work, I would like to stress the importance of providing comprehensive and reactive support. The potential community interested in xTEA is somehow significant and I expect that forthcoming requests of assistance can be easily diverted by providing detailed, step by step instructions. A minimum would be something of the level of detail given in the MELT manual, though I encourage the authors to provide additional tutorials and guidelines to promote the use of their method. I also appreciate the availability of a docker repository, however I was not able to locate it and find related documentation.

- Regarding the comparison of performances with MELT, I understand the choice of the authors to focus on this tool rather that the (quite significant) number of other methods available. I would appreciate, possibly as supplementary material, a rationale on discarting some of the more recent and/or less popular methods. This information is crucial to guide potential users and educate on the strengths and weaknesses of the methods they are seeking for their projects.

- Because the documentation is not complete yet, it is not clear for me a this point how the different data and methods can be used and combined practically. I also recommend to add to

the documentation a summary of the results regarding parameters such as sequencing technology and depth, read quality and other source of variation in the data.

- I really appreciated the benchmarking performed by the authors using manually curated TE on the HG002 genome. Undoubtedly, xTEA shows its relevance to identify insertion in 'difficult' regions such as heterochromatin and other repeat-rich regions. However, there are also a few but significant PCR benchmark datasets available for individuals of the 1000 Genome Project (PMIDs: 28465436, 32075552 for example). I think that comparison of xTEA genotypes to PCR data will be a strong addition to the manuscript. In particular, while most TE genotyped by PCR show a strong signature of Hardy-Weinberg equilibrium, in my experience, tools such as MELT tend to underestimate the real number of heterozygotes (non-ref insertions), leading to slightly biased estimators such as the imbedding coefficient. Given the new genotyping method, I am curious to see how does xTEA perform on this aspect.

- Finally, it was not clear to me whether xTEA computational performances are improved compared to MELT. It seems to me from figS.8 that xTEA can quickly use much more memory than MELT for a marginal improvement in time/cpu. Can the author provide for comparable dataset

'Comment/Questions'

- One main limitation of xTEA is that it does not handle reference insertions. Though this is a justified decision of the authors, I believe that if published their tool will reach a much larger audience than those interested in non-reference TE insertions. In particular I suspect that reference polymorphic insertions may segregate in average at higher frequency in the population and may encompass functional or regulatory polymorphisms. Does the authors plan on expending the method to reference insertion? Is there technical or methodological challenges regarding these loci?

- SVA seem to be the hardest elements to type. xTEA performance are comparable to MELT suggesting a real challenge with short reads. Do you have evidence of improved typing for SVA using long reads?

- There are multiple variables set by default in the xTEA algorithm that can be user changed. It will be useful to have some documentation regarding the range of parameters and their effects (read ratio in genotype training set, --nclip, --cr, --nd, --nfclip, read ratio in genotype training set, etc...). Could the authors provide some general guideline?

- xTEA is human-specific though I anticipate that it can be useful for other species and other TEs. What are the current caveats that the author anticipate would occur if xTEA is used with other species?

- Though the authors described quite precisely how their method is innovative, I would be interested to see more technical details about how they tested and selected parameters and

implemented their method (as an informatics/mathematical problem) in supplementary material. I believe that sharing these information will improve reproducibility and add value to the publication.

Looking forward to review a revised version of the manuscript,

Best regards,

Clément Goubert

RESPONSE TO REVIEWERS

We thank the reviewers for recognizing the value of our work and for their detailed assessment. The comments we received were very useful, and addressing them has significantly improved the quality of our manuscript. We have carried out many additional analysis—the number of Supplementary Figures in the manuscript increased from 9 to 16, and the number of pages in the Supplementary Methods from 13 to 22. We first summarize the key points, and then provide more detailed point-by-point responses.

SUMMARY

Reviewer #1 requested a comparison with other general-purpose SV long read callers and a long-read TE insertion caller PALMER, as well as additional experiments on error-prone long reads (a previous generation of the PacBio platform). In addition, the reviewer wanted more clear documentation on github with a bioconda package.

Reviewer #2 also asked about how xTea compares to existing general purpose short and long read SV callers, including whether it works well on error-prone long reads. The reviewer requested that we use a different benchmark dataset. The reviewer also asked for a better evaluation of somatic L1 calling for tumor samples, a better introduction for the HERV section, and a more detailed technical illustration of the machine learning-based genotyping module.

Reviewer #3 gave several suggestions, including more detailed documentation on xTea and information about the performance of xTea in terms of speed and memory cost. The reviewer also requested additional comparison with other TE insertion callers and to benchmark the performance of the genotyping module with existing PCR data.

Here is a summary of our responses:

- Comparison with other SV callers: We benchmarked two popular short read SV callers, Delly and Manta, on TE insertion calling. The results showed their low sensitivity in detecting TE insertions. We also added a comparison with three long read SV callers (Sniffles, SVIM and CuteSV), which showed lower sensitivity or lower specificity than xTea. Our comparison with a long read TE insertion caller PALMER also showed a higher false positive rate for PALMER than xTea. One important point to note is that "calling TEs" is not simply to find breakpoints but also to annotate them accurately—some general SV callers can find the breakpoints but do not annotate properly.
- xTea evaluation for error-prone long reads: We added xTea performance evaluation for PacBio and Nanopore error-prone long reads.
- Evaluation on more benchmark data: We provided a more detailed description of how we generated the phased TE insertion benchmark dataset. In addition, we performed xTea evaluation using another sample with high confident L1 insertion benchmark data and found consistent results. We also evaluated the

performance of the xTea genotyping module using PCR-based benchmark data from a published study and confirmed high accuracy of xTea genotyping.

- Comparison with TraFiC on more tumor samples: Our previous comparison with TraFiC was on one lung tumor sample. We performed a more comprehensive comparison using 15 colon cancer samples and found that xTea showed a significantly higher sensitivity with a similar specificity than TraFiC.
- More detailed documentation: We added a detailed documentation about xTea installation, parameter setting and output. We also created a bioconda package of xTea for easy installation.
- Github repo for reproducible results: We created a new github repo (https://github.com/parklab/xTea_paper) to host commands, scripts and parameters for reproducing the results in the paper.

POINT-BY-POINT RESPONSES

Reviewer #1

In this paper, Chong Chu et al nicely describe a method for detecting mobile elements of various types from both long and short read sequencing data. This method, xTea, is a helpful addition to the array of techniques currently available to call these events in both short and long read data. The biggest improvements here are the application to both long and short read data, the clear annotation of even LTR elements, and the annotation of the elements that are called. The authors use their method to call insertions in an extensively characterized dataset, HG002, and generated haplotype resolved calls in this genome. They find great improvements in accuracy and precision, especially for L1s, with long read data, and their method performs quite well. This is especially true, given the nature of long reads, for L1s in repeat regions, such as the centromere and those that accompany structural rearrangements. The methods and entire paper are well written. A previously developed tool does have the benefit of using long read data, and PALMER comparisons would actually likely benefit the authors manuscript.

We would like to thank the reviewer for recognizing the value of our work and for the useful suggestions to extend the analysis.

The major critiques I have of this paper are the following;<br>**1.1** The authors mention PALMER, which calls L1s in long read data, but do not compare their method to this existing tool.<br>**1.2** Additionally, they only utilize HiFi reads; it would be nice to see how their cigar/split read approach performed with less accurate CLR reads. Furthermore, the comparison to PALMER, if the authors improve their user friendliness, would be astounding.<br>**1.3** The multithreading is already an improvement. A bioconda package would be a larger improvement.<br>**1.4** The ability of the method to be adapted to novel genomes other than human given a library of mobile element consensus sequences could be an important improvement to the existing technique.

1.1 In the revised version, we ran PALMER on the HG002 HiFi data, and compared its performance with that of xTea on the same benchmark data. We used two groups of PALMER calls in the comparison: “PALMER raw”, which are all the reported calls, and “PALMER HC”, which are high confident calls with the author-recommended cutoff in the PALMER github README. Our new Fig. S8 (also copied here) shows the comparison results.

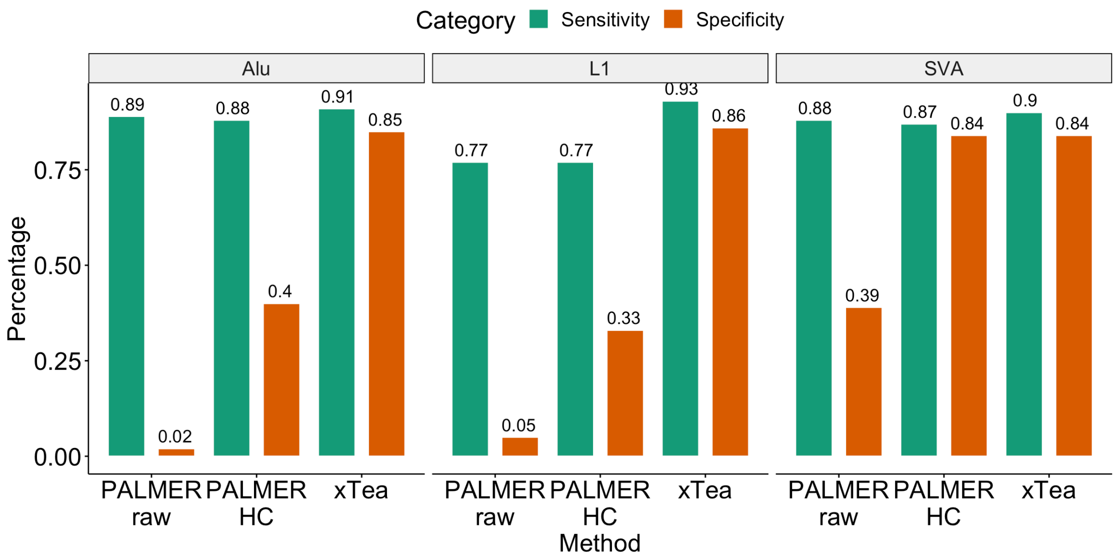

The results show that xTea outperforms PALMER on both sensitivity and specificity across all three TE families. Notably, PALMER reports many false positives, resulting in low specificity especially for Alu and L1. In addition, PALMER was extremely slow to run, taking >30 days without producing any output on this ~35X PacBio HiFi data. So, we had to run PALMER for each chromosome separately to obtain this comparison result.

1.2 We added the xTea evaluation results for PacBio CLR and Oxford Nanopore reads from HG002. Both datasets have ~45X coverage. We revised Fig. 2b to include the comparison as follows.

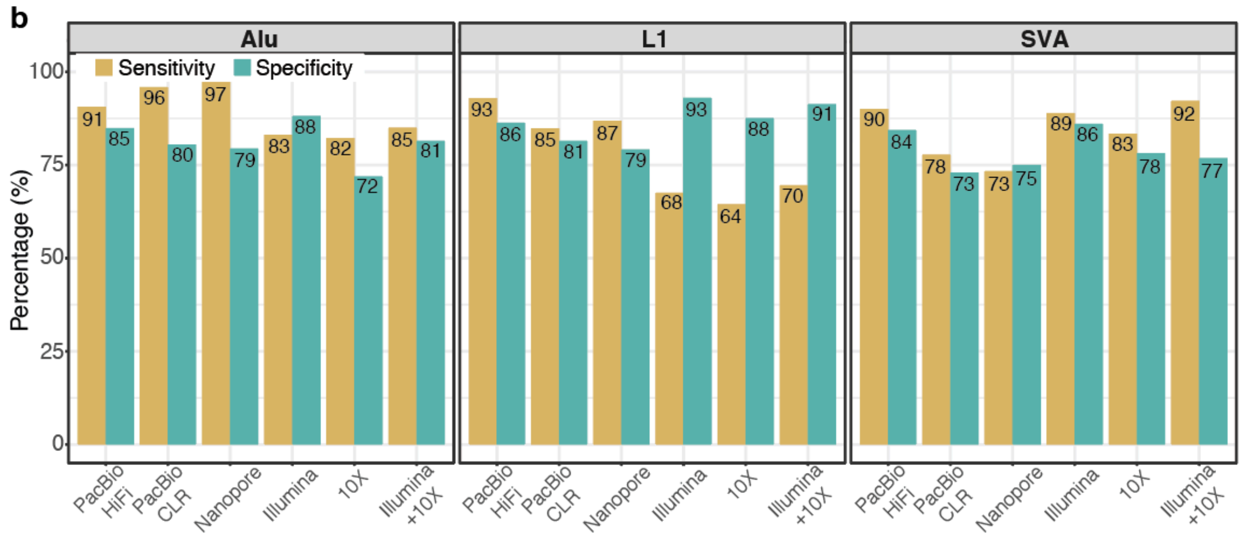

The xTea performance was comparable for PacBio CLR and Nanopore long reads. Compared to PacBio HiFi reads, the sensitivity of the less accurate long reads was lower for L1 and SVA. The major reason is that xTea only reports TE insertions that have the insertion sequences fully assembled, and many insertions detected in the error-prone long reads failed in the sequence assembly and/or final calling step of aligning the flanking regions to the contigs because of the high error rate. In addition,

long VNTR repeats within SVA may have caused difficulty in the assembly. We only report TE insertion breakpoints for Illumina short reads without reporting fully assembled insertion sequences, and so this likely explains the better SVA performance for Illumina short reads than for less-accurate long reads.

1.3 We have produced a bioconda package for xTea and updated github.

1.4 xTea was initially designed to be used for any species. However, to improve specificity, we later added a TE-type-specific filter for Alu, L1, and SVA. Thus, users can run xTea on a new TE type of a species other than human, as long as they skip the very last filtering step. We appreciate the reviewer's suggestion on making the xTea available for other species, but we do feel that designing of additional filtering steps to minimize false positives and a thorough evaluation will take a significant amount of work that is the beyond the scope of the current manuscript. We do have a great interest in further improving xTea for non-human species in the future.

2) The confusion matrix for HG002 calls could use some filling in. I would appreciate knowing the # of false positive calls from xTea.

Thank you for the comment. We added the details of our benchmark results in supplementary Table S1-S3 (copied below).

Method	Coverage	F1	Sensitivity	FDR	TP	FP	FN
Illumina	60X	0.855	0.830	0.118	1125	151	230
Illumina+10X	120X	0.832	0.850	0.185	1152	262	203
10X	60X	0.767	0.821	0.281	1113	434	242
Nanopore	45X	0.875	0.974	0.206	1320	342	35
PacBio CLR	45X	0.875	0.959	0.196	1299	316	56
PacBio HiFi	30X	0.876	0.906	0.151	1227	219	128

Tab. S1: Performance of xTea in detecting Alu insertions on different sequencing platforms on sample HG002.

Method	Coverage	F1	Sensitivity	FDR	TP	FP	FN
Illumina	60X	0.782	0.675	0.070	133	10	64
Illumina+10X	120X	0.790	0.695	0.0867	137	13	60
10X	60X	0.743	0.645	0.124	127	18	70
Nanopore	45X	0.8280	0.868	0.208	171	45	26
PacBio CLR	45X	0.831	0.848	0.185	167	38	30
PacBio HiFi	30X	0.895	0.929	0.137	183	29	14

Tab. S2: Performance of xTea in detecting L1 insertions on different sequencing platforms on sample HG002.

Method	Coverage	F1	Sensitivity	FDR	TP	FP	FN
Illumina	60X	0.874	0.889	0.140	80	13	10
Illumina-10X	120X	0.838	0.922	0.231	83	25	7
10X	60X	0.806	0.833	0.219	75	21	15
Nanopore	45X	0.742	0.733	0.250	66	22	24
PacBio CLR	45X	0.753	0.778	0.271	70	26	20
PacBio HiFi	30X	0.871	0.900	0.156	81	15	9

Tab. S3: Performance of xTea in detecting SVA insertions on different sequencing platforms on sample HG002.

> 3) The Docker platform is nice to have, but their script on github needs work. A bioconda package would make the tool more widely use-able, given the dependencies for the code. Additionally, the README file does not currently contain all of the packages/versions and parameters required to run the tool, and does not state whether the tool can perform in python 3. Further issues include hardcoded email addresses in the shell script that are particular for running the job on slurm. The script does seem to be scheduler dependent, and that is a large issue for submitting the jobs on another HPC that uses Torque/MOAB.

Thank you for the suggestions to improve the usability of xTea. We have created an xTea bioconda package and revised the README file to describe all the dependent packages/versions. xTea is now compatible with both python 2 and 3. We removed the script header information as they are different for different systems, and users can add that based on their own HPC system.

> 4) The long read work flow had missing files that required import from the short read version (rep_lib_annotation.tar.gz). Additionally, there are many parameters that are not explained, and need adequate commenting and sections in the README or help pages.

The same TE library file (rep_lib_annotation.tar.gz) is used for both short and long reads modules. We have updated the README to describe this and explained how to set the parameters as the reviewer requested.

> 5) The comparison between long read and short read data are a bit unfair; the genotype data in MELT are not robust, and TypeTE is far improved. The use of insertion calls from Long read callers like PBSV or SVIM to overlap with their tool would give a better picture of the unique knowledge gained by using xTea over long read SV caller annotation, given that short read data are notoriously bad for identification of insertions. A comparison with PALMER would be even more useful.

The comparison among different sequencing technologies is more to show their advantages and disadvantages in calling TE insertions.

Following the suggestion, we tried to run TypeTE for comparison but found that TypeTE works only for Alu insertions. We also failed to set up and run it despite multiple attempts, due to the many dependencies with other packages. There was no docker or bioconda package for TypeTE.

We did run three long read SV callers, CuteSV, Sniffles and SVIM, on the same HG002 HiFi data. Fig. S11 (also copied here) shows the number of SVs reported by each tool. SVIM called ~10 times more SVs compared to CuteSV and Sniffles, likely indicating a very large number of false positives.

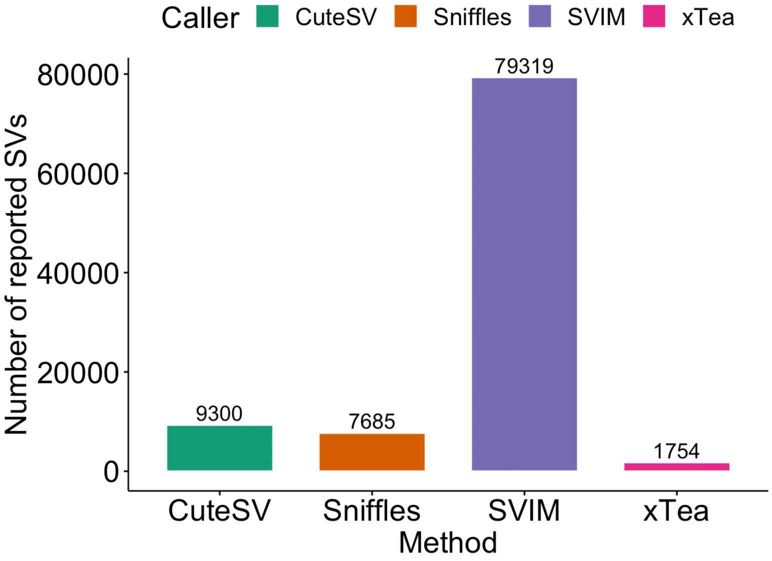

We compared the sensitivity of the three tools with xTea using the same benchmark data on HG002. As shown in Fig. S12 (also copied here), the results show that both CuteSV and Sniffles show low sensitivity, especially for L1 and SVA. It is worth noting that:

1) CuteSV and SVIM only report insertion breakpoints, and do not assemble the insertion sequences, while Sniffles and xTea report both.
2) As described in Fig. S11, SVIM reports ~10 times more SVs than the other two SV callers. So, although its sensitivity is higher, it has too many false positives.
3) Reviewer #2 also raised the similar concern (comment #1). We have more comparison with general short reads SV callers and discussions regarding TE insertion annotation there.

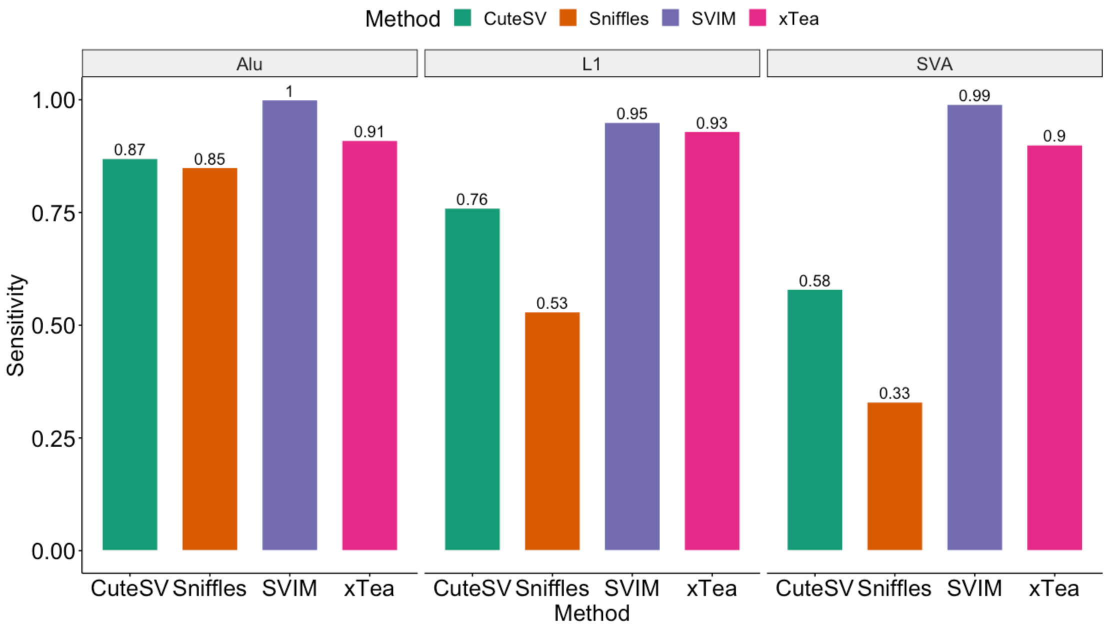

In summary, the comparison with general long read SV callers emphasizes the importance of transposon-specialized callers, such as xTea, that assemble and annotate TE insertions to achieve high sensitivity and specificity.

Reviewer #2

Transposable elements (TEs) frequently occurs in the human genome, and TE insertions are involved in human diseases when the insertion disrupts human genes. The authors developed a novel computational tool (xTea) for identifying transposable element insertions from short-read, linked-read and PacBio long-read whole-genome sequencing data. The main advantage over existing approaches is that xTea can be applied to long-read data, which is expected to have highly sensitivity and specificity to detect TEs, compared to conventional Illumina short-read sequencing. They also created a high-confidence set of TE insertions for the GIAB HG002 genome. The authors benchmarked xTea on this data set and a large pedigree data set. The authors showed that xTea outperformed MELT and TraFiC-mem.

xTea identifies TE insertions using methods that is also used by general insertion callers (e.g. discordant read pairs, clipped/split reads, insertion within read alignment, local assembly), with some optimizations. The authors compared xTea to some other TE insertion callers. However, it would be interesting to compare xTea with a general-purpose insertion detection pipeline. For example, a general-purpose SV detection pipeline can use Manta/Pindel/fermikit to call insertions for short reads and SMRT-SV/PhasedSV/Sniffles to call insertions for PacBio long reads, and use Repeatmasker to annotate the repeats. The reason why it is important is that as a user of long-read sequencing data, I will never use an existing TE caller (as they are not really designed for PacBio/Nanopore data) for TE calling. There are a number of SV callers that are published in the past a few years, each claiming superior performance, and most (if not all) of them allows the identification of TE insertions (it requires slight post-processing of results by annotation by repeatmasker). A comparison to these tools on long-read data would be more informative, even though these tools were general purpose SV calling tools.

Thank you for the constructive suggestions. We have performed three analyses to address the issues raised.

1) Comparison with general SV callers for Illumina short reads

We ran two popular general SV calling methods, DELLY and Manta, on the same WGS short reads for HG002 and compared their sensitivities with xTea's using the same benchmark data. As presented in Fig. S10 (also shown below), both DELLY and Manta showed much lower sensitivity in calling TE insertions than xTea for all three TE families. Furthermore, for these detected by the general SV callers, most were not annotated as TE insertions but as other types of SVs, such as translocation and duplication, due to short reads not uniquely mapping to exact source loci. To overcome this low sensitivity and incorrect annotation of general SV callers, the 1000 Genomes Project (Sudmant et al. 2015) and gnomAD-SV database (Collins et al. 2020) used a specialized caller for TE detection, MELT, to construct catalogues of polymorphic TE insertions in addition to general SV callers.

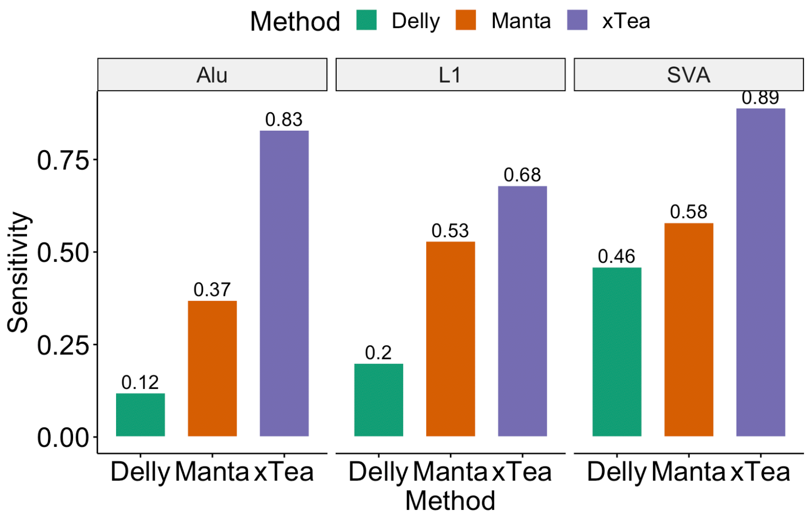

2) Limitation of using existing long read SV callers with RepeatMasker annotation

Some of the long-read SV callers, including Sniffles, assemble insertions of any kind. The reviewer is correct that users can run such an SV caller and then RepeatMasker to annotate TE insertions. However, the approach will have following serious limitations:

- RepeatMasker annotation generally works well for simple TE insertions, such as Alu and canonical L1 insertions; however, it cannot accurately annotate non-canonical insertions or SVA insertions. For SVA, it creates multiple separate annotations for almost all SVA components, e.g., the hexamer and the VNTR region within SVA are always annotated as “simple repeat”. In Tab. S5 (also showed below), we show a more complex example of an SVA insertion, called CH10_SVA, that was fused with the MAST2 gene and is still actively creating new insertions in the human genome. The table shows information from RepeatMasker output (except for the column “Label”). This single SVA insertion was annotated as 8 subfamilies, thus we cannot tell the family of this insertion

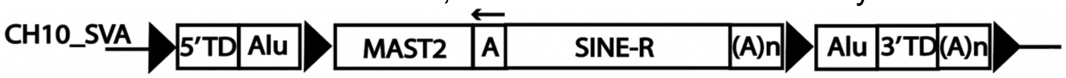

only by RepeatMasker annotations.

Label	Start	End	Orientation	Sub-family	Family
5’ TD	23	110	C	MIR	SINE/MIR
Fusion	185	321	+	AluSc	SINE/Alu
	533	575	+	(GCC)n	Simple_repeat
SVA	697	1459	+	SVA_F	Retroposon/SVA
	1086	1902	+	SVA_F	Retroposon/SVA
	1523	2692	+	SVA_F	Retroposon/SVA
Fusion	2717	3016	+	AluSp	SINE/Alu
	3097	3117	+	(A)n	Simple_repeat
3' TD	3349	3589	+	L1ME3G	LINE/L1
	3590	3682	+	L1MA10	LINE/L1
	3683	3761	+	L1ME3G	LINE/L1
PolyA	3774	3794	+	(A)n	Simple_repeat

- RepeatMasker cannot properly annotate insertions with transductions, which comprise >15% of L1 and >15% of SVA insertions. Because the transduction sequences can be masked to one or more repeat segments of any TE type or TE subfamilies. With these non-unique family annotations from RepeatMasker, we cannot tell which "family" these insertions are.
- RepeatMasker cannot annotate those complex insertion events, such as TE insertion-promoted duplications.

In contrast, xTea has a stand-alone module to annotate TE insertions in detail, such as TE subfamily, target site duplication (TSD), polyA tails, internal SVs and transductions.

3) Comparison with long read general SV callers
Please refer to our response to comment #5 by Reviewer #1 who asked the same question. Long read SV callers showed lower detection accuracy or reported only insertion breakpoints without assembled insertion sequences, when tested on the same benchmark HG002 dataset.

In conclusion, our comparison emphasizes a critical need of specialized computational methods, such as xTea, to detect, construct, and annotate TE insertions for both long and short sequencing data.

> The paper showed benchmarks for PacBio HiFi reads, but claimed that xTea also works for regular long-read sequencing platforms (e.g. PacBio raw reads and Oxford Nanopore long reads). Intuitively the main difference would be that regular long-read data have higher basecalling error rates, but at the same time can offer longer read length to enable more accurate alignment and SV calling. Since all the "benchmark genomes" are now extensively sequenced by multiple technologies, it would be important to benchmark these genomes using conventional PacBio data as well as Nanopore data, so that readers have a good sense of the performance of the algorithms under different scenarios. These data (for example HG002 genome) are high-coverage public datasets on multiple technical platforms.

Thank you for the suggestion. The same point was raised by Reviewer #1. Please see our response on page 5, section 1.2.

> For similar reasons, it would be interesting to see if you can validate the centromeric L1 using data from other platforms (e.g. ultra-long nanopore reads, which is available for HG002). The ability to computationally validate specific predictions using a different (somewhat orthogonal) technical platform would yield confidence on the predictions on a challenging call in an extremely challenging area of the genome.

Thank you for the suggestion of utilizing the data from orthogonal platforms to validate centromeric ghost L1s we reported. We agree that it would provide confidence for our calls to some degree. However, we thought it would actually be more effective to utilize a recently released telomere-to-telomere assembly of the CHM13 cell line (Miga et al.

2020). Thus, we ran xTea on the PacBio HiFi WGS data for CHM13 and identified 13 centromeric ghost L1s: 12 and 1 full-length L1s with flanking regions masked as Alpha and Beta satellite repeats, respectively. We checked whether the ghost L1s we predicted could be found in the CHM13 v1.0 assembly (https://github.com/nanopore-wgs-consortium/CHM13) by aligning the assembled ghost L1s with their flanking regions back to the CHM13 v1.0 assembly using minimap2 (Li 2018), and confirmed all of the 13 events in the assembly.

We also attempted to evaluate how many centromeric full-length L1s were present in the assembly. First, we ran RepeatMasker on the CHM13 v1.0 assembly and selected full-length (>5950bp) L1s flanked by centromeric satellite repeats (>5000bp) at least on one side of the breakpoints. Surprisingly we found 114 such L1 copies as shown in Tab. S8 (also shown here).

LINE-1 sub-family	Number of copies
L1PA5	3
L1PA4	17
L1HS	12
L1PA2	29
L1PA3	53

Even with long reads, different centromeric L1s cannot be distinguished from each other when they have the same long centromeric flanking repeats. Due to this limitation, xTea's ghost L1 detection module can only call full length L1 copies whose flanking sequences could be distinguished. Thus, we checked how many L1s out of 114 copies from the assembly are unique in terms of their flanking sequences by grouping them according to their flanking sequences. We obtained 19 unique copies because 96 copies carried the same flanking repeat sequences. Given this, we think 13 ghost L1s xTea produced is reasonable. To clarify this point, we revised "copies" to "group of copies" in the text. We have pushed the detailed RepeatMasker output of these 114 copies to the paper github repo https://github.com/parklab/xTea_paper.

Additionally, GIAB themselves also released their own benchmark SV set on HG002 ("The final benchmark set contains 12745 isolated, sequence-resolved insertion and deletion calls =50 base pairs (bp) discovered by at least 2 technologies or 5 callsets"). It would not be difficult to simply annotate their calls and compare to your calls. Note that in their Figure 3 of GIAB, they already showed the expected peaks for Alu insertions and deletions near 300 bp and for full-length LINE1 insertions and deletions near 6000 bp. I am also a bit confused when reading this paper, since it seems that the authors somehow used the GIAB calls (~9.9K calls in a specific version of release plus assembly-based calls) to build their benchmarking data set, rather than using the xTea to help build a better benchmarking set. Nevertheless, if the authors want to claim that their "1,642 haplotype-resolved high-confidence TE insertions (1,355 Alu, 197 L1 150 and 90 SVA insertions; Fig. S5)" is of better quality than what the GIAB has released to the public domain, some evidence needs to be shown and some

comparisons need to be done. I also have the same concern of not using Nanopore data here in xTea analysis (they presented Illumina, 10X and HiFi data); note that the GIAB benchmarking set was built using 5 callsets.

We thank the reviewer for raising this issue. We had indeed used the GIAB data to create our benchmark dataset as we described in the section “Creation of a haplotype-resolved benchmarking dataset”. We revised the description to improve clarity. We first combined all GIAB insertion calls larger than 50bp (v0.6) (Zook et al. 2020) with all >50bp insertion calls from the haplotype-resolved assembly (Li et al. 2020) into one call set. As none of these insertions were annotated for TEs, we ran RepeatMasker and selected insertions that had at least one segment annotated as LINE-1, Alu or SVA. Then, we did manual inspection for each of them through both IGV and RepeatMasker output, and selected 1,642 TE insertions as our final benchmark.

Note that SVs from these two sources are called by two different approaches:

- The GIAB benchmark set was created using 5 different methods, all of which are alignment-based.
- The haplotype-resolved SV set was called by the phased-assembly.

Fig. S5 (also copied here) shows the data source of the 1,642 final TE insertion benchmark set. 1,611 of the final benchmark TE insertions (98%) were phased calls from the haplotype resolved assembly, and 1,408 TE insertions (86%) were called by both methods.

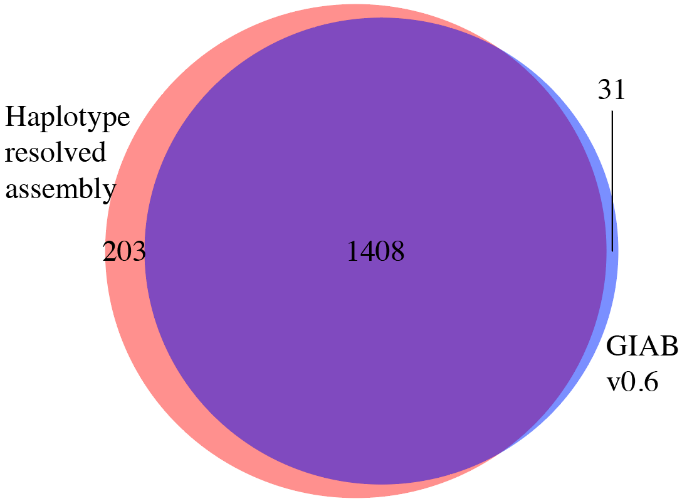

The reviewer mentioned Figure3 in the GIAB paper. We would like to note that the TE insertion annotation (Figure 3) in the GIAB paper was based on MELT, a TE insertion caller for short Illumina reads, not long reads, so it would not be sufficient to serve as a TE insertion benchmark set.

To the best of our knowledge, the HG002 benchmark TE insertion set we created is the most comprehensive and of high-quality, and thus will be a useful resource for many other studies.

In Figure 2, I do not understand the rationale of combining Illumina and 10X Genomics Illumina data together and present the results. In principle, if xTea can combine results from multiple technologies and generate consensus calls, it would be ideal to add a real long-read technology based caller in generating such results. This is important because HiFi alone actually achieves better performance than the “Illumina-10X” combination in Figure 2b.

The hybrid calling mode does not generate a simple combined set of the final calls from each platform. Instead, it integrates intermediate read-level information supporting each insertion (e.g., discordant reads and clipped reads) from each platform as if they are derived from one bam file. Different platforms have their own advantages and disadvantages. For example, the cloud-based alignment for linked reads may generate better alignment in some repetitive regions and provide the linked information for phased assembly of TE insertions. But their lower read quality may cause more non-specific clipped and discordant reads than Illumina short reads. Accordingly, we observed the improved TE detection of the hybrid calling and recommend it when the data from both platforms are available for the same sample. We do agree with the reviewer that HiFi alone already achieves better performance, but here we want to show the comparison among different platforms, including hybrids, to give a better guide for users who may have one or more types of data.

In addition to HG002, the authors should also create the same benchmarked xTea data sets on other GIAB genomes, such as HG001, and the parents of HG002. All these genomes are extensively sequenced by multiple sequencing technologies, including Illumina sequencing and PacBio sequencing and Nanopore sequencing, and the data are publicly available. Part of the reason why people use HG002 nowadays is the availability of parental information, so such analysis should yield additional insights into the performance of any software tools to call mutations, as well as the intrinsic properties of specific type of mutations in terms of de novo mutations rates and stability in specific genomic regions.

Is We initially used HG001 (NA12878) for our benchmark because of several early released PacBio long reads data for the sample. We used TE calls from long reads to evaluate methods for short reads. However, we later decided to use HG002 for two major reasons:

- Lack of high quality TE insertion benchmark data that can be used to evaluate the performance of tools working on both short and long reads;
- The cell line HG001 has genome instability (private email conversation with Heng Li), which may cause the sequencing data from different labs to be different. Thus, recent benchmark datasets for different studies are of from HG002.

Nonetheless, we ran xTea on the HG001 PacBio HiFi data, as requested. We obtained 208 L1 insertions and compared the result with a published L1 call set for the same sample by Zhou et al. (2020), which consists of 203 L1 insertions using PALMER PacBio CLR reads along with manual inspection. The comparison is reported in Fig. S9

now. There are 168 common insertions, and 40 xTea-specific and 35 PALMER-specific insertions. Since PALMER finds only L1 insertions, we could not compare the Alu and SVA calls.

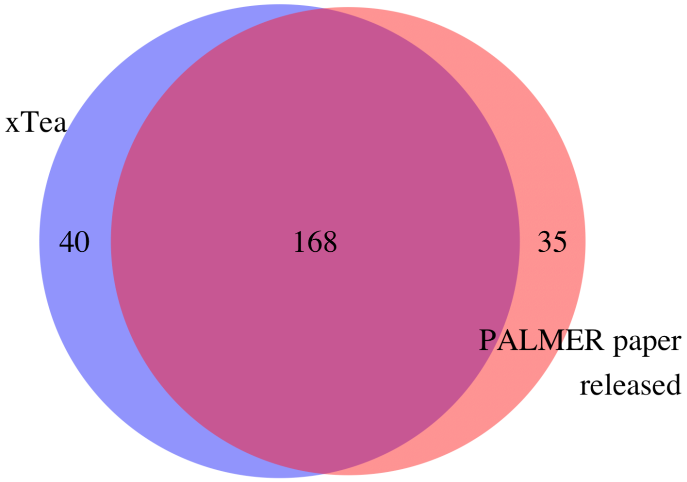

We do appreciate the point that a trio should be helpful for validation. However, from our analysis of PacBio and Nanopore WGS data from 20 individuals, we infer that *de novo* insertions are rare—with an expected rate of 1 per 20 births (Feusier et al. 2019) —so, we expect the insight we could gain by analyzing a trio would be limited.

> I think the ability to find somatic L1 insertion is an interesting topic to address, given the known role of re-activated L1 insertion in tumor progression. However, the current paper used only one single lung cancer sample, and compared to a different method called TraFiC-mem here. I am not sure what is the purpose here exactly and why this particular sample is used (it does not look like it is a well known benchmarking sample with a set of known somatic TE, and MELT is not used here either). Given that a number of cancer cell lines with matched germline cells are sequenced by PacBio or Nanopore (in addition to Illumina), I would think it makes more sense to perform comparison on something where multiple orthogonal technologies have been used to generate data, to evaluate performance of the method on somatic mutations. The ability to generate more calls in a Venn diagram does not necessarily translate to improved performance of xTea over TraFiC-mem.

We agree with the reviewer that this section was weak. The problem was that there is no high-quality benchmark dataset for somatic TE insertions, and so every single call from a tumor sample had to be manually reviewed, making it difficult to scale it up. As the reviewer mentioned, there are multiple long reads WGS datasets for some cancer cell lines, such as K562 and SK-BR-3. Unfortunately, they are not cancer types in which high somatic TE insertions have been reported.

We selected the one lung cancer sample because it was reported to have a large number of somatic L1 insertions. The reason we did not include MELT in the comparison was that MELT was designed to detect germline insertions and does not

provide a module to call somatic events. MELT also has limited capability in detecting transduction events, which occur frequently in cancer genomes.

To strengthen this section, we have now compared somatic TE insertion calls from xTea and TraFiC-mem from the top 15 colon cancer samples from the PCAWG consortium based on their reported somatic L1 insertion counts (Rodriguez-Martin et al. 2020). For each insertion candidate called by xTea and/or the PCAWG study, we performed manual inspection using the IGV browser (Robinson et al. 2011). Manual inspection is by no means a perfect method, but in most cases, we were able to effectively distinguish true positives from false positives while trying to be as unbiased as we possibly could. Our analysis summarized in Figure 3f and 3g confirmed that xTea showed higher sensitivity than TraFiC-mem at a comparable precision.

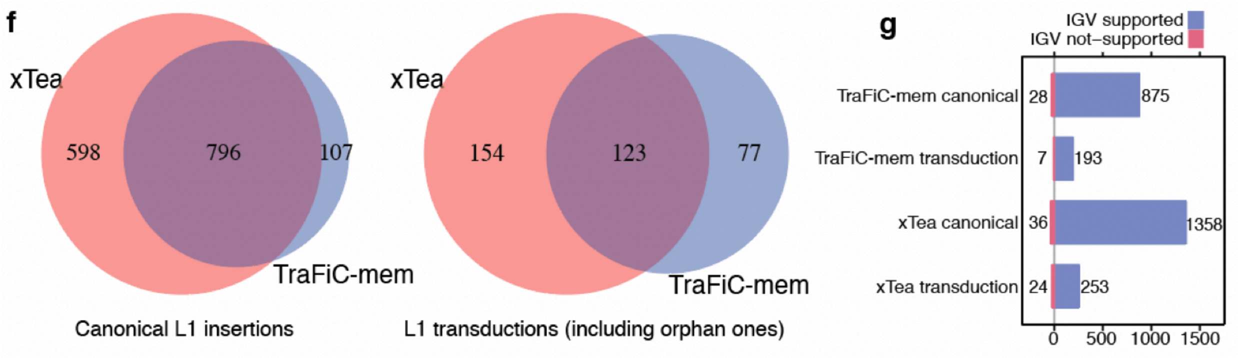

Around Page 17 line 320, the authors described their efforts in finding novel HERV. I am not familiar with this field but I found it to be interesting. The Figure 5c and 5d are not particularly informative and I cannot tell what information it intends to present: among the six known HERV and six novel ones, I do not see which is which, and the text does not mention it either. I feel that this entire section can benefit from some additional background introduction to HERV, so that readers have a better understanding of current knowledge on them, and explain how/why current Nanopore-based genome assembly cannot be used to examine and identify more HERVs (from what I read, if there are de novo genome assembly then it is straightforward to directly call HERV from the assembly itself).

We appreciate the reviewer's comments on this section. We have added more background and introduction on HERVs to help the readers better understand this section:
"Endogenous retroviruses (ERVs) are derived from exogenous retroviruses that are integrated into the host genomes. A full-length (proviral) ERV is comprised of an internal protein-coding region flanked by two long terminal repeats (LTRs). Several human ERV (HERV) families have been observed to be associated with several diseases, including several cancers, neurological and autoimmune diseases (Gröger and Cynis 2018; Küry et al. 2018; Bannert et al. 2018; Desai et al. 2017; Tokuyama et al. 2018). Because of the sequence homology, LTR-LTR recombination will result in the deletion of the internal coding sequence. For the same proviral HERV, if recombination only happens in some samples, it will result in "dimorphic HERV", where the reference genome is a

solo LTR but may be proviral HERV in individuals. Many of these complex events of different HERV subfamilies, for instance HERV-K and HERV-H, have been reported from short paired-end reads analysis. However, short reads can be used to check the two tail sides of an event, but they do not provide the full structure; short reads also do not provide information for those events in repetitive or complex regions."

In Fig. S16, we showed the steps to call dimorphic HERV events with xTea. We agree with the reviewer that if there are good assemblies, these changes could be called from the assembly itself. What we showed in Fig. 5c and 5d are not something Nanopore-based genome assembly cannot do, but, to the best of our knowledge, they haven't been done from long reads.

I do not really see what is the "Machine learning-based TE insertion genotyping for short reads". No description was given in the paper, in either Methods or Results section (except an illustrative panel in Figure 1), except that 14 features are used for each candidate. As a methods paper, details on the actual methods are needed. The reported 99.7% accuracy is also somewhat concerning as it is a bit too high, yet an examination of the statement shows that they have >1.3 million TE sites yet only 19K non-TE sites in the analysis which is a highly unbalanced set.

In Methods, there was a section titled "Machine learning-based TE insertion genotyping for short reads" and Fig. S15 "PCA for TE insertion genotype features and feature importance" had the list of features. We have now added more details to this section.

The reviewer is correct that the training data was unbalanced with more TE sites than non-TE sites. It is mainly because we genotyped TE insertion candidates that passed the cutoff filters and were called by xTea; thus in the training dataset, most TE insertions would have genotype "0/1" or "1/1", and only a small fraction of false positive calls would have "0/0." Given the similar ratio of TE genotypes expected in real genotyping situations, we think the reported accuracy of 99.7% from the testing dataset is likely to be generalized to real data.

Some additional minor issues are noted below:

In page 4, line 57. This is wrong. The long reads can be much longer than 15 kb. In fact, in most Nanopore sequencing data that we use today, even the N50 length itself is already a lot higher than 20kb.

We have revised the text as follows:
"such as PacBio and Oxford Nanopore long reads create >10-15 Kbp reads"

In page4, line 59. “To date, PALMER21 is the only tool designed for long reads, but it is limited to detection of canonical L1 but not Alu or SVA insertions.” This is not a fair statement. Almost all long-read SV callers can detect TE (users have to annotate the SV calls to know if they are L1 or Alu), but they are general purpose callers, not something specifically designed for TE calling.

We have revised the statement to “*specifically designed for TE insertion calling on long reads*”.

In page 12, figure 3a. It is better to show both recall, precision.

Now, we show the following plots for sensitivity and specificity in Fig. S6.

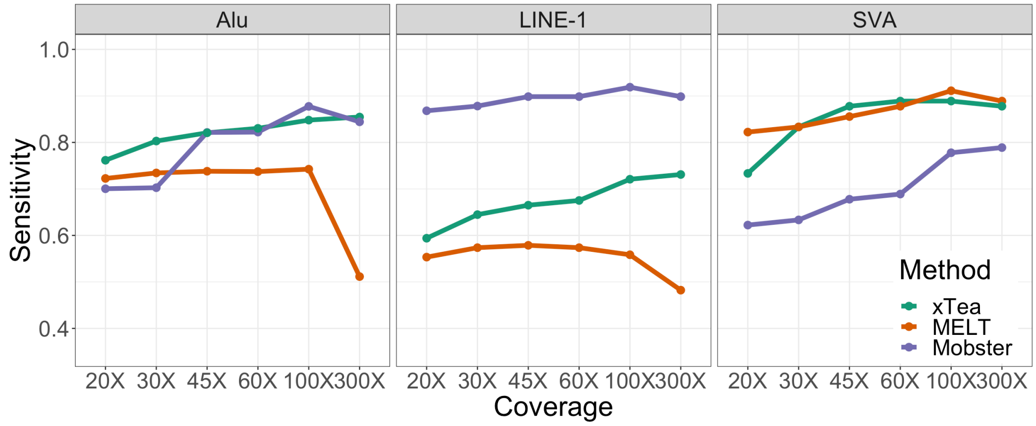

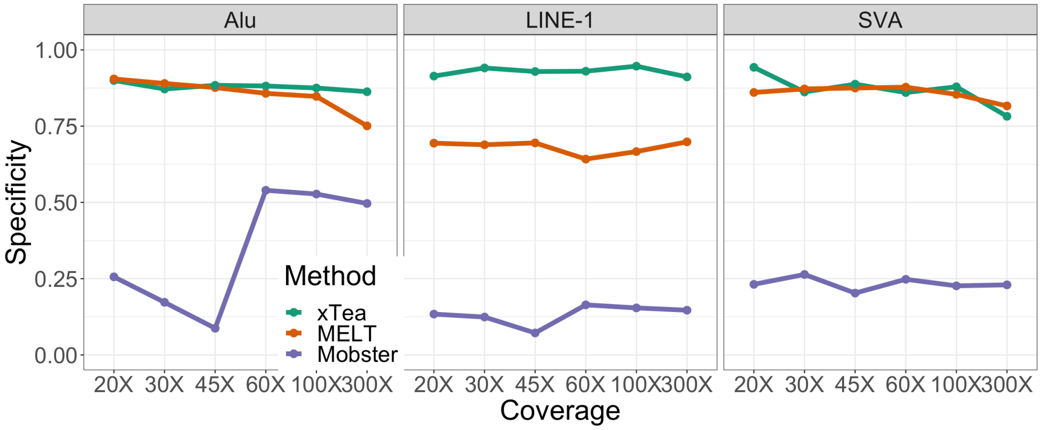

In page 22, line 435. The author removed genomic regions with extremely high molecule coverage (> 250X) in the linked-read sequencing data. However, 250X is not extremely high for molecule coverage (I think you mean the coverage of reconstructed molecules/fragments from linked reads). In a typical 30X linked-read sequencing data, the average molecule coverage is about 150X.

The reviewer was correct for our definition of molecule coverage. The molecule coverage cutoff is a user-specified parameter of xTea, with a default value of 250X. The default works reasonably for the normal sample data we tested. We would suggest a higher cutoff for tumor sample data.

In line 366. The polymorphic TE insertions are not necessarily more recent the TEs in the reference genomes.

We revised the text to read "*it is important to annotate polymorphic TE insertions, which are generally more recent and may play an important role in regulating the host gene expression*".

In figure S6, the points with genotype "0" and "1" are not well separated. I didn't see "~90% of the points can be clustered". It would be better if you try more methods, such as tSNE.

The training data is large (996,714 points). Here we randomly selected 9,000 insertions (3,000 for each genotype) and clustered them with tSNE. Genotype 0, 1, and 2 are in red, green and blue respectively. Note, we defined those false positive ones from xTea's output as genotype "0" when we build the training set, which means they all at least have some "clip" and "discordant pair" support. Thus, for some cases, some features are shared between "0" and "1" in our training data. That's why some are not well "classified" from tSNE view.

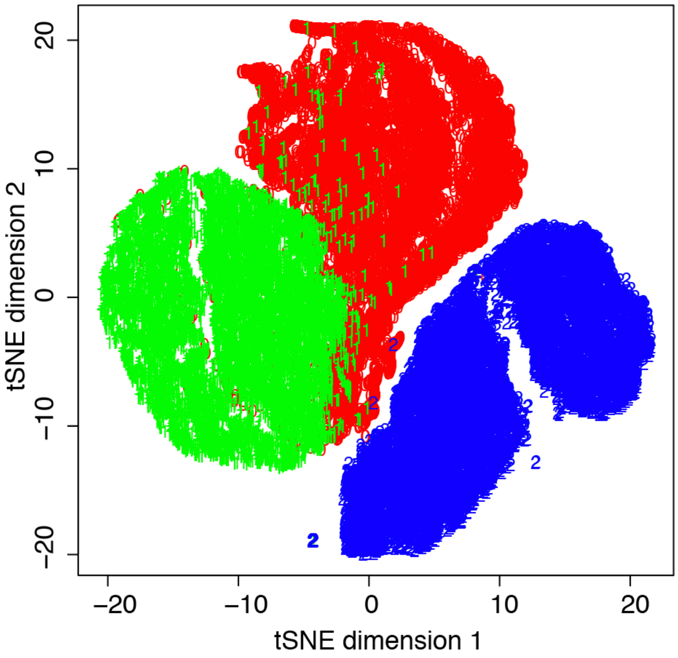

General comments: since the method can be applied to both short and long read data, it is best to indicate in the legend of each figure what data sets are used for the results. This helps readers understand the results better, as some figures are purely based on short-read sequencing.

We added the description of datasets used in the legend of each figure.

Reviewer #3:

I have now reviewed the manuscript of Peter J. Park and co-authors entitled "Comprehensive identification of transposable element insertions using multiple sequencing technologies". The authors present a new version their bioinformatic tool TEA, xTEA. xTEA detects and genotypes non-reference human transposable element insertions. xTEA allows users to use genomics data from a wide range of sequencing technologies including short, short-tagged and long read sequencing.

The paper has a strong focus on human health and the potential of their method for gene therapy.

The method is original and provide a significant enhancement of the currently published alternatives (see 'Strengths'). In my opinion, the methodology is accurate and incorporate state-of-the-art tools and algorithms. As of its suitability for publication in Nature Communications, I will likely support this decision if the authors respond to the points listed in 'Weaknesses'. I also include a list of questions or remarks that I will be grateful for the authors to reply, even though these issues do not dispute the reliability of the method (see 'Comments/Questions').

'Strengths'

I my opinion, xTEA provide a significant enhancement of the current methods available for human TE data analysis. In particular:
- Ability to use and combine short, long and synthetic long reads (Ill. 10X) of the most popular sequencing technologies.
- Significantly higher sensitivity and genotyping accuracy for L1 elements which are historically hard to detect and genotype accurately.
- Implementation of a new, original genotyping algorithm for TE based on machine learning
- Higher or equivalent overall performances with most likely competitor method (MELT 'discovery')
- Detection of ERVs, and genotyping of full-proviral vs solo-ltr HERV insertions.
- Detection and classification of TE-related structural variants
- New high-quality TE genotype reference dataset.

We thank the reviewer for recognizing the value of our work.

'Weaknesses'

- My principal critique concerns the current availability of user support. Although the software and basic instructions are available online (via GitHub), the current manual is in my opinion too short and does not allow a new user to reproduce seamlessly the experiments shown in the paper. While the depiction of the pipeline is very clear in the manuscript, the instructions online did not allow me to perform a complete analysis and combine the different modules at the time of the review. Though this is a time-

consuming work, I would like to stress the importance of providing comprehensive and reactive support. The potential community interested in xTEA is somehow significant and I expect that forthcoming requests of assistance can be easily diverted by providing detailed, step by step instructions. A minimum would be something of the level of detail given in the MELT manual, though I encourage the authors to provide additional tutorials and guidelines to promote the use of their method. I also appreciate the availability of a docker repository, however I was not able to locate it and find related documentation.

We should have done a better job in our first submission. We have now updated the xTea README to include more detailed instructions. We also provide a bioconda package and a docker image.

- Regarding the comparison of performances with MELT, I understand the choice of the authors to focus on this tool rather that the (quite significant) number of other methods available. I would appreciate, possibly as supplementary material, a rationale on discarting some of the more recent and/or less popular methods. This information is crucial to guide potential users and educate on the strengths and weaknesses of the methods they are seeking for their projects.

Thank you for the suggestions. We have tried to run three additional TE insertion callers on HG002 Illumina short reads: RetroSeq, TEMP and Mobster. However, we failed to get the final results for RetroSeq and TEMP (note that the github sites for both tools have not been maintained for the last 5 years). We added the performance of Mobster in Fig. 3a (also copied here).

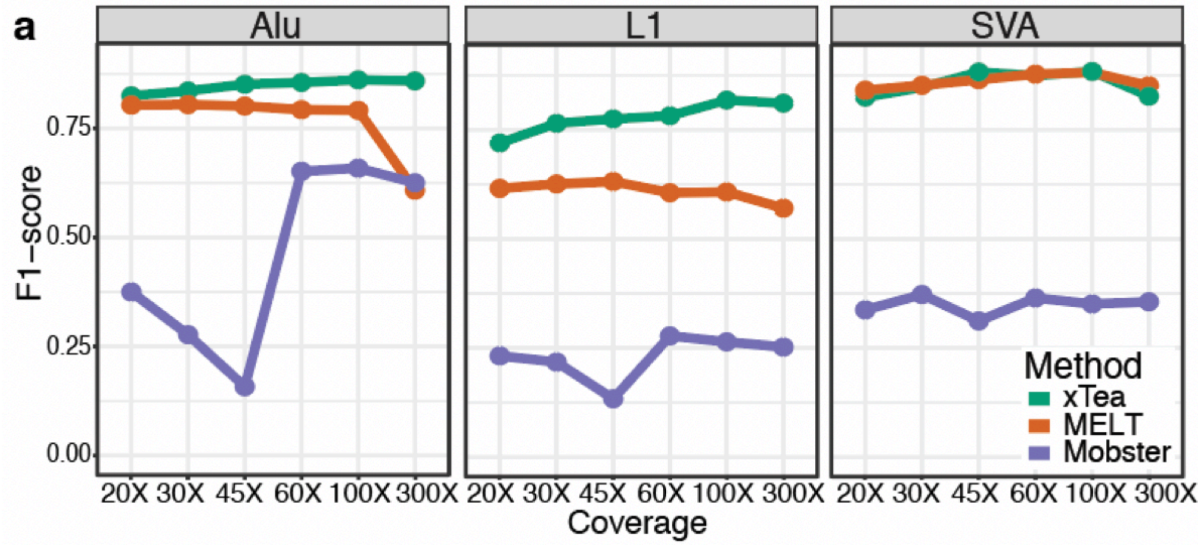

- Because the documentation is not complete yet, it is not clear for me a this point how the different data and methods can be used and combined practically. I also recommend to add to the documentation a summary of the results regarding parameters such as sequencing technology and depth, read quality and other source of variation in the data.

We created a new github repository (https://github.com/parklab/xTea_paper) to host commands, scripts, and intermediate results to allow users to reproduce our analysis.

- I really appreciated the benchmarking performed by the authors using manually curated TE on the HG002 genome. Undoubtedly, xTEA shows its relevance to identify insertion in 'difficult' regions such as heterochromatin and other repeat-rich regions. However, there are also a few but significant PCR benchmark datasets available for individuals of the 1000 Genome Project (PMIDs: 28465436, 32075552 for example). I think that comparison of xTEA genotypes to PCR data will be a strong addition to the manuscript. In particular, while most TE genotyped by PCR show a strong signature of Hardy-Weinberg equilibrium, in my experience, tools such as MELT tend to underestimate the real number of heterozygotes (non-ref insertions), leading to slightly biased estimators such as the imbedding coefficient. Given the new genotyping method, I am curious to see how does xTEA perform on this aspect.

As suggested by the reviewer, we evaluated the performance of xTea using a PCR benchmark dataset (Payer et al. 2017). This dataset reported PCR validation results at 145 Alu sites for 90 samples from the 1000 Genomes project including 45 high-coverage (~30X) samples. So, we downloaded these 45 samples from the 1000 Genomes Project data portal (https://www.internationalgenome.org/data-portal/data-collection/30x-grch38) and ran xTea on them to call TE insertions and genotype them. We compared xTea genotypes with the PCR benchmark dataset (the study only reported benchmark for Alu), converting hg18 coordinates to hg38, and obtained the following table (Tab. S4). The detailed comparisons are provided in the xTea paper Github repo (https://github.com/parklab/xTea_paper/tree/main/Genotype_cmp):

- 19 sites are overlapping between xTea calls and the PCR benchmark and have PCR-genotype as “0/1” or “1/1” in at least one sample;
- 27 PCR genotypes do not have any information, thus are removed;
- Thus, in total 19*45-27=828 genotypes;
- In the end, 659, 126, and 43 sites are PCR-genotyped as “0/0”, “0/1”, and “1/1” respectively;
- For xTea, if no insertion is reported for the given site, then the genotype of this site for this sample is “0/0”.

**Genotype**		**PCR**		
		0/0	0/1	1/1
**xTea predicted**	0/0	659	0	0
	0/1	0	126	**11**
	1/1	0	0	32

The results show highly consistent genotypes between xTea and the PCR benchmark data, except for the 11 sites that xTea genotyped as heterozygous (0/1) but the PCR data showed homozygous (1/1). These 11 genotypes may have been incorrectly predicted by xTea, but it is also possible that the genotypes from PCR are imprecise.

- Finally, it was not clear to me whether xTEA computational performances are improved compared to MELT. It seems to me from figS.8 that xTEA can quickly use much more memory than MELT for a marginal improvement in time/cpu. Can the author provide for comparable dataset

To improve both sensitivity and specificity, xTea inevitably incorporated more extensive read utilization and extra filtering steps than MELT. However, by implementing multiple thread support, xTea dramatically reduced per-core memory requirement, ~2GB per-core memory with 3 hrs 50 min run time with 16 cores as shown in Fig. S3 (previously S8; shown below) and the following table. MELT does not support multiple threads and requires 18 GB memory to analyze the same ~45X WGS sample.

Methods	Cores	Memory	Time
xTea	1 core	3,011MB	30hrs
	2 cores	5,395MB	22hrs
	4 cores	7,576MB	16hrs
	8 cores	15,112MB	7hrs
	16 cores	19,257MB	3hr 50mins
MELT	Single thread	18,024MB	5hrs 13mins

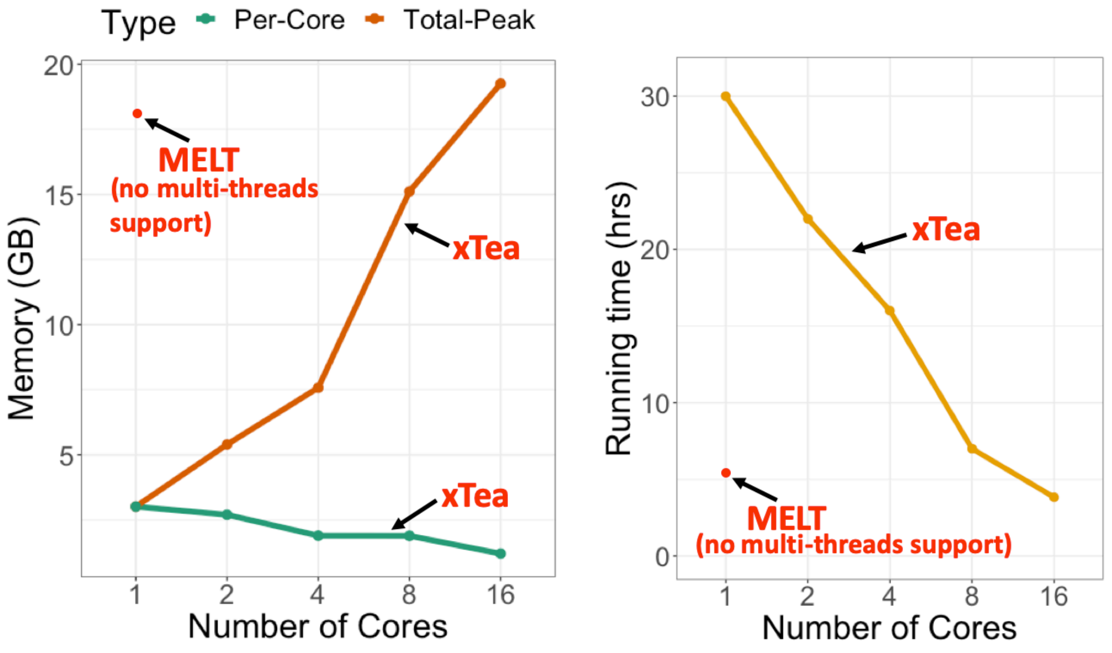

'Comment/Questions'

- One main limitation of xTEA is that it does not handle reference insertions. Though this is a justified decision of the authors, I believe that if published their tool will reach a much larger audience than those interested in non-reference TE insertions. In particular I suspect that reference polymorphic insertions may segregate in average at higher frequency in the population and may encompass functional or regulatory polymorphisms. Does the authors plan on expending the method to reference insertion? Is there technical or methodological challenges regarding these loci?

We appreciate the reviewer's suggestion. For now, we do not plan to identify reference insertions simply because it can be done effectively using existing callers. Polymorphic reference copies are technically deletions when we analyze a sample without the copy, so we expect general SV callers that can call deletions would be able to detect such events effectively when coupled with proper TE annotation using RepeatMasker.

- SVA seem to be the hardest elements to type. xTEA performance are comparable to MELT suggesting a real challenge with short reads. Do you have evidence of improved typing for SVA using long reads?

As the length of most of the SVA insertions are much shorter than the long reads, long reads do show better performance in calling SVA insertions. However, still many are not called accurately. We carefully examined these missed SVA insertions from long read data, and found that the bottleneck was not in the discovery step but in the assembly step. Several of those missed ones showed clear features supporting the insertions, for example enough clipped reads support, to be included in the initial raw calls. But they failed in the contig assembly or flanking region alignment steps, and thus were excluded in the final calls. We plan to try different assembly strategies to improve SVA detection in future work.

- There are multiple variables set by default in the xTEA algorithm that can be user changed. It will be useful to have some documentation regarding the range of parameters and their effects (read ratio in genotype training set, --nclip, --cr, --nd, --nfclip, read ratio in genotype training set, etc...). Could the authors provide some general guideline?

We apologize for the insufficient description of the xTea parameters. We have updated the github description. Users do not need to provide any cutoff values for parameters by default, as they are automatically set based on the estimated read depth. But for tumor samples, users may need to set the tumor purity level if it is substantially different from the default one (0.45) to achieve better performance. Although the automatic parameter configuration was internally benchmarked to optimize xTea performance, xTea also provides flexibility for advanced users to set up their own parameters as now described in more detail in the github repo.

- xTEA is human-specific though I anticipate that it can be useful for other species and other TEs. What are the current caveats that the author anticipate would occur if xTEA is used with other species?

Thank you for the question. We addressed this question also raised by Reviewer #1 in section 1.4 in page 5.

- Though the authors described quite precisely how their method is innovative, I would be interested to see more technical details about how they tested and selected parameters and implemented their method (as an informatics/mathematical problem) in supplementary material. I believe that sharing these information will improve reproducibility and add value to the publication.

We have added more detailed information for the method in supplementary and more description about the parameter setting in the two github repos.

REVIEWER COMMENTS

Reviewer #1 (Remarks to the Author):

The authors have addressed many of the reviewer comments and suggestions. There are a few things that are remaining issues for this manuscript.

1) The bioconda package, for either short or long reads, does not run. An error is encountered; no information is given. For a method to be published, it must be able to be implemented outside of the development environment. This issue was encountered in a laboratory that regularly implements difficult software/pipelines

2) There are still typos. Perhaps a quick check will help; line 640, for instance, says "gnome" where genome was intended.

3) The issue with regards to xtea only being developed for human data was not addressed. I realize this may be outside the scope of this manuscript.

Reviewer #2 (Remarks to the Author):

The authors have made an effort to address the comments that were raised in the previous version of the manuscript.

In particular, for the initial (probably more serious concerns), they have performed three analyses to address the issues and presented data and figures supporting their results. These results are convincing.

Additionally, I mentioned a place where the authors described their efforts in finding novel HERV yet this aspect did not include sufficient amount of background information for a typical reader to understand. I think they made an effort to rewrite this section to increase its readability and to increase its interpretability.

For my comments about figure S6,where the points with genotype “0” and “1” are not well separated, the authors provided a response with a figure. Three genotypes are represented by three different colors, respectively. The presentations are improved than before, perhaps you can incorporate the new results into one of the figures or sup figures instead.

I also checked the author’s response to other reviewers’ comments, and they all look reasonable to me.

RESPONSE TO REVIEWERS

We thank the reviewers for their detailed assessment of our first-round revision. The remaining issue was that there was a problem running xTea with bioconda. We believe we have resolved this issue with bioconda package upgrades, as verified by independent tests by several people.

POINT-BY-POINT RESPONSES

Reviewer #1

> The authors have addressed many of the reviewer comments and suggestions. There are a few things that are remaining issues for this manuscript.
>
> 1) The bioconda package, for either short or long reads, does not run. An error is encountered; no information is given. For a method to be published, it must be able to be implemented outside of the development environment. This issue was encountered in a laboratory that regularly implements difficult software/pipelines

We traced this error to the fact that xTea requires some packages that need to be installed first and that some of them should be of specific (or later) versions. It will cause error if different versions are installed.

To resolve the issue, we have set the specific versions for the packages in bioconda. Four of our lab members who do not work on transposable elements have tested the latest bioconda version (v0.16.0), and no errors were reported. In addition, we have updated the README in github to make the dependencies clearer.

> 2) There are still typos. Perhaps a quick check will help; line 640, for instance, says "gnome" where genome was intended.

We have fixed this and have carefully gone through the manuscript again.

> 3) The issue with regards to xtea only being developed for human data was not addressed. I realize this may be outside the scope of this manuscript.

We feel that generalizing xTea to other species is out of scope for the current manuscript. We have multiple extensions and applications to pursue, including expansion of xTea to work on other species.

Reviewer #2

> The authors have made an effort to address the comments that were raised in the previous version of the manuscript.

In particular, for the initial (probably more serious concerns), they have performed three analyses to address the issues and presented data and figures supporting their results. These results are convincing.

Additionally, I mentioned a place where the authors described their efforts in finding novel HERV yet this aspect did not include sufficient amount of background information for a typical reader to understand. I think they made an effort to rewrite this section to increase its readability and to increase its interpretability.

For my comments about figure S6,where the points with genotype “0” and “1” are not well separated, the authors provided a response with a figure. Three genotypes are represented by three different colors, respectively. The presentations are improved than before, **perhaps you can incorporate the new results into one of the figures or sup figures instead**.

I also checked the author’s response to other reviewers’ comments, and they all look reasonable to me.

We would like to thank the reviewer for recognizing the revisions we have made in the first round. Regarding the comment about incorporating the figure, we had already done that—the new genotype classification figure is Fig. S15(c) in our previous revision.

REVIEWERS' COMMENTS

Reviewer #1 (Remarks to the Author):

I greatly appreciate the review and bug fixes of the bioconda package; we have not tested this in the laboratory as of yet, but given this revision and the other edits to the paper, I now find the manuscript ready for publication.
This work is both timely and interesting. I appreciate the author's thorough responses to all of the reviewer's comments.
